# Causes of death after testicular cancer diagnosis: a US population-based analysis

**DOI:** 10.1186/s12894-023-01309-3

**Published:** 2023-09-02

**Authors:** Zhongyuan Wang, Baochao Li, Jiajun Xing, Zixuan Gong, Aiming Xu, Zengjun Wang

**Affiliations:** Department of Urology, First Affiliated Hospital of Nanjing Medical University, No. 300, Guangzhou Street, Nanjing, 210029 Jiangsu Province China

**Keywords:** Testicular cancer, Cancer survivorship, Causes of death, Surveillance, Epidemiology, and End Results (SEER), Database

## Abstract

**Background:**

After the introduction of cisplatin-based chemotherapy, the survival time of testicular cancer (TC) patients has improved dramatically. However, the overall risk of death in patients with TC remains significantly higher than in the general population. The aim of this study was to assess and quantify the causes of death after TC diagnosis.

**Method:**

In total, 44,975 men with TC in the United States diagnosed and registered by the Surveillance, Epidemiology, and End Results (SEER) database during 2000 to 2018 were studied. In this study, standardized mortality rates (SMRs) were calculated for each cause of death in TC individuals and further analyzed in strata according to age and race.

**Result:**

Of the included participants, 3,573 (7.94%) died during the follow-up period. The greatest proportion of deaths (38.20%) occurred within 1 to 5 years after diagnosis. Most deaths occurred from TC itself and other cancers. For non-malignant conditions, the most common causes of death within 1 years after diagnosis were accidents and adverse effects (53, 4.75%) followed by diseases of heart (45, 4.04%). However, > 1 years after diagnosis, the most common noncancer causes of death were heart diseases. Results of stratified analysis show that non-Hispanic White TC participants have a lower SMR (0.68, 95% CI, 33.39–38.67) from Cerebrovascular Diseases than the general U.S. population.

**Conclusions:**

Although TC remains the most common cause of death after TC diagnosis, other non-TC causes of death represent a significant number of deaths among TC men. These findings help TC survivors understand the various health risks that may occur at different follow-up periods.

**Supplementary Information:**

The online version contains supplementary material available at 10.1186/s12894-023-01309-3.

## Introduction

Testicular cancer (TC) is the most common malignancy in young men, with an all-age incidence of up to 10 per 100,000 [1]. In 2021, it is estimated that there will be 9,470 new cases of testicular cancer in the United States, with approximately 440 deaths resulting from the disease, according to the latest projections. Fortunately, survival rates for testicular cancer in developed regions have exceeded 90% [2], which is attributable to improvements in chemotherapy (i.e., cisplatin) [3, 4].

Considering the prolonged life expectancy of young patients with TC, it is becoming increasingly important to assess non-TC causes of death (COD) in TC participants. And it has been shown that radiation and chemotherapy for TC patients leads to increased second malignancy and cardiovascular mortality [5–9]. In addition, Fung et al. [10] found increased mortality due to pneumonia and influenza in TC patients compared to the general population. While Fossa et al. observed an elevated mortality risk from fibrosis and pneumonia [11].

In the present study, we provide the largest and most updated U.S. population-based analysis to date of the risk of non-cancer mortality in TC participants over 15 years of follow-up and explore the association between these non-cancer COD and demographic characteristics. We also provide an overview of the change as compared to the general U.S. population in the risk of each non-cancerous death for TC participants across the time period of diagnosis.

## Method

### Data source

We selected the incidence-SEER Research Plus Data, 17 Registries, Nov 2021 Sub (2000–2019) dataset using the SEER*Stat software (version 8.3.9), which cover approximately 28% of the general US population between 2000 and 2019 (www.seer.cancer.gov). SEER data are anonymized and are considered as nonhuman subject research. Thus, the need for Ethics Committee approval was waived.

### Study population and outcomes

**Fig. 1:**
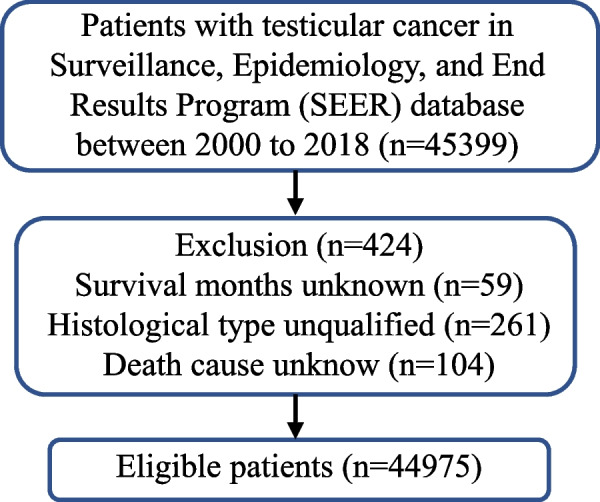
Flow diagram illustrating recruitment of patients

To minimize selection bias, we included all individuals diagnosed with TC and histologically confirmed between 2000 and 2018 in the SEER registry. There were 45,399 TC men in the above database, of whom 261 lacked histological validation, 104 lacked causes of death, and 59 lacked survival time. Excluding these participants, a total of 44,975 TC men were included in this study (Fig. 1).

Causes of death were obtained using the SEER Cause of Death Recode, which was established based on the WHO International Statistical Classification of Diseases and Related Health Problems, Tenth Revision [12, 13]. Under the category infectious diseases, we included other infectious and parasitic diseases including HIV, pneumonia and influenza, septicemia and tuberculosis. Under the category other cause of death, we included congenital anomalies, homicide and legal intervention and symptoms, signs and ill-defined conditions.

We reported percentages of deaths (among TC individuals) within each follow-up period and percentages of each cause of death (among deaths in TC individuals) within each follow-up period. To measure the risk of a specific cause of death in TC participants, we used the standardized mortality ratio (SMR). SMR is the ratio of the number of observed deaths to the number of expected deaths. The number of observed deaths refers to the number of TC participants who died from a specific cause of death in a given time frame, and the expected represents the number of people expected to die from the same cause of death in a demographically similar population (adjusted for year of diagnosis, age, sex, and race) in the same time frame. In the current study, SMR represents the difference in the risk for a specific cause of death after TC diagnosis compared to the general US population. We further stratified SMRs by age, race or ethnicity, and time after diagnosis. Besides, we used the data from 2000 to 2018 to adjust and match the year of TC diagnosis when comparing causes of death in participants who had TC with that of general population [14].

### Statistical analysis

In our study, the competing risk model was used to compare the overall prognosis of TC patients in SEER treated with different therapies. In this model, after categorizing all patients into three groups of non-testicular cancer deaths, competing events, and review events, the Fine-Gray algorithm was used to calculate and compare mortality rates. We use cumulative incidence function (CIF) to display the probability of occurring non-TC death and competing events, and use Gray's test to estimate the CIF differences between subgroups [15, 16].

To better understand the epidemiology of TC, we calculated age-adjusted TC incidence rates and annual percentage changes from 1975–2018 using SEER*stat and Joinpoint [15], and use multi-phase regression to fit the change of annual incidence [16]. We also used SEER*Stat software to calculate SMR and 95% confidence intervals (CI). An increased risk of death was considered when the observed number of deaths was greater than the predicted number of deaths and the *P* value was less than 0.05. All tests were two-tailed and *p*-values less than 0.05 were considered statistically different.

## Results

### Patients characteristics

**Fig. 2:**
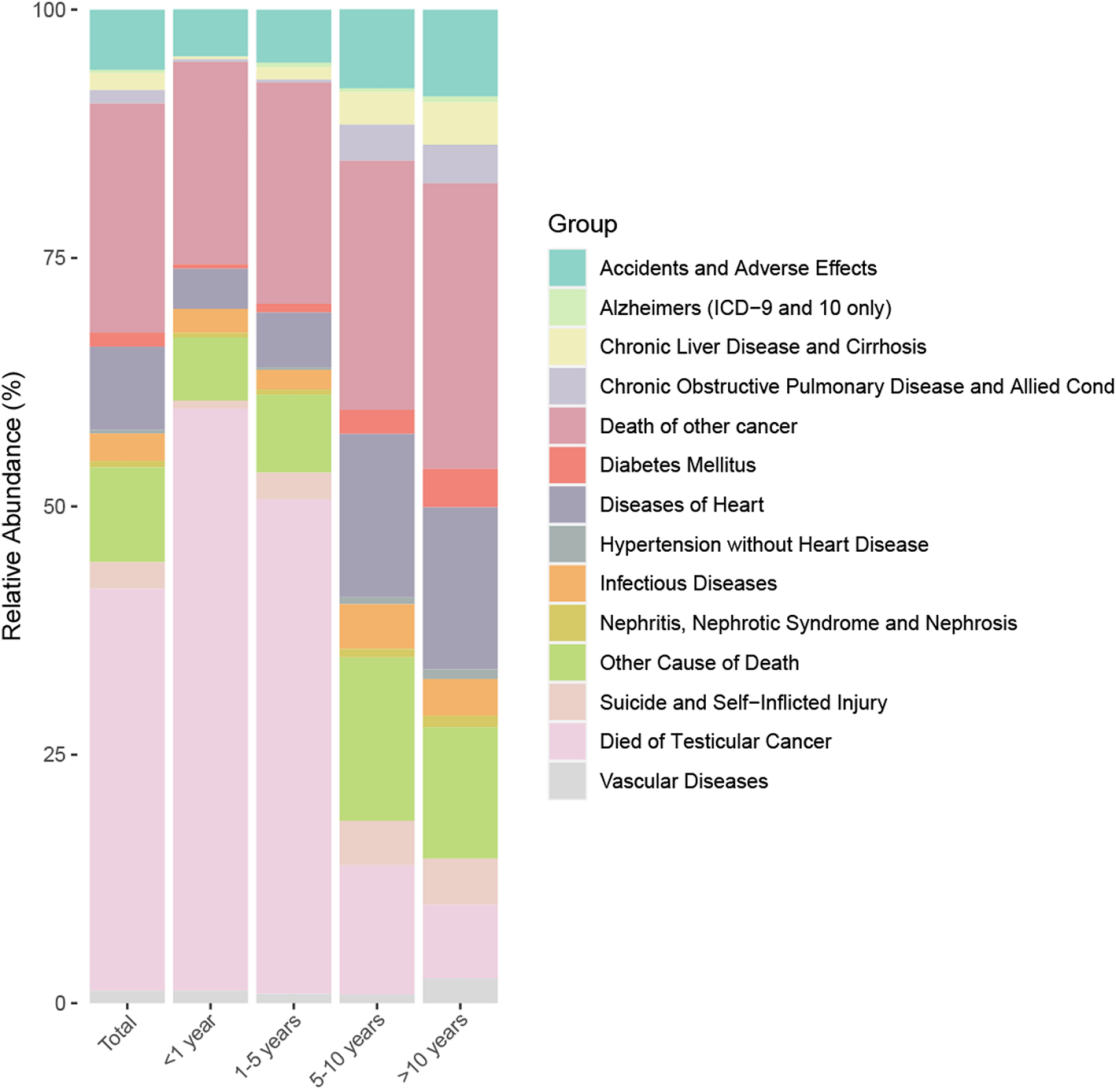
Causes of death in each latency period following testicular cancer diagnosis. Notes: SMR: Standardized Mortality Ratio

Our study included 44,975 participants with TC, of them 3,573 (7.94%) died. Most (32.08%) were between 30 and 40 years old and median age at diagnosis was 36.2 years, 31.53% were between 20 and 30 years old, and 18.91% were between 40 and 50 years old, while 11.80% and 5.68% were over 50 years old or under 20 years old, respectively. Among the deaths, the range of the interval from diagnosis to death of the TC patients was from zero month to 227 months and the median age of death at follow-up ranged from 40 to 50 years, with the most common age of death being over 50 years (Table 1). The highest number of death (1,265; 39.29%) occurred within 1 to 5 years after TC diagnosis. Meanwhile, 932 deaths (28.94%) occurred within < 1 year after TC diagnosis, 531 (16.49%) occurred within 5 to 10 years, and 492 (15.28%) occurred > 10 years. Overall, TC itself was the most common cause of death, of which 92.18% of deaths occurred within the first 5 years of diagnosis. The most common non-TC deaths were other cancers (824; 23.06%), diseases of heart (299; 8.37%), accidents and adverse effects (217; 6.07%) and infectious diseases (99; 2.77%). The Fig. 2 shows the proportions of different causes of death according to time of death after TC diagnosis.

**Table 1:** Baseline characteristics of patients with testicular cancer and patients who died according to the time of death after diagnosis

	Total	Alive	Dead				
			Total	< 1 year	1–5 years	5–10 years	10 + years
Number	44,975	4,1402 (92.05)^b^	3,573 (7.94)	1,115 (31.20)	1,365 (38.20)	578 (16.18)	515 (14.41)
Follow-up Time (months)	96.89 ± 67.45^a^	100.97 ± 66.89	49.55 ± 54.67	4.53 ± 3.61	28.77 ± 13.88	87.78 ± 17.73	159.22 ± 25.37
Age							
< 20	2555 (5.68)	2406 (5.81)	149 (4.17)	42 (3.77)	78 (5.71)	18 (3.11)	11 (2.14)
20–30	14,180 (31.53)	13,376 (32.31)	804 (22.50)	283 (25.38)	376 (27.55)	85 (14.71)	60 (11.65)
30–40	14,429 (32.08)	13,607 (32.87)	822 (23.01)	251 (22.51)	323 (23.66)	118 (20.42)	130 (25.24)
40–50	8503 (18.91)	7785 (18.80)	718 (20.10)	206 (18.48)	246 (18.02)	131 (22.66)	135 (26.21)
50 +	5308 (11.80)	4228 (10.21)	1080 (30.23)	333 (29.87)	342 (25.05)	226 (39.10)	179 (34.76)
Race							
White	40,413 (89.86)	37,239 (89.94)	3174 (88.83)	961 (86.19)	1215 (89.01)	530 (91.70)	468 (90.87)
Black	1390 (3.09)	1193 (2.88)	197 (5.51)	83 (7.44)	59 (4.32)	31 (5.36)	24 (4.66)
Other race	2209 (4.91)	2016 (4.87)	193 (5.40)	66 (5.92)	91 (6.67)	16 (2.77)	20 (3.88)
Unknown	963 (2.14)	954 (2.30)	9 (0.25)	5 (0.45)	0 (0.00)	1 (0.17)	3 (0.58)

^a^Mean + SD

^b^N (%)

### Competing risk analyses

**Fig. 3:**
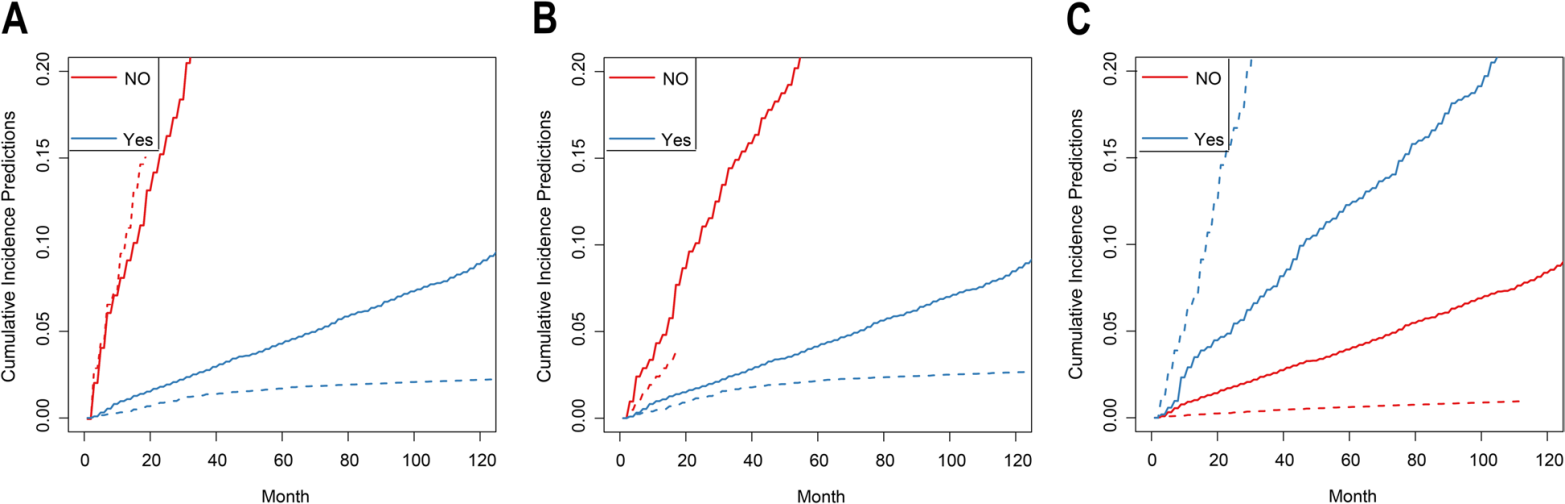
**A** Cumulative incidence curves of cause-specific death according to surgery. **B** Cumulative incidence curves of cause-specific death according to radiotherapy. **C** Cumulative incidence curves of cause-specific death according to chemotherapy. Notes: SMR: Standardized Mortality Ratio

By conducting Fine-Gray analysis, we found that different treatment modalities significantly influence the overall prognosis of patients with TC. Figure 3 shows the CIF curves for different treatment modalities. The CIF for patients who did not receive surgery or radiotherapy were higher, while the opposite was true for patients who received chemotherapy. The CIFs of non-TC deaths within 10 years after diagnosis were higher for patients in stage II and III than those in stage I (Fig. 3 and Supporting Fig. 2). Table 2 presents the specific CIF values.

**Table 2:** Competing risk analysis in patients with testicular cancer

Variables	Gray’s test	*p*-Value	Cumulative incidence function		
			12-months	60-months	120-months
AJCC Stage	55.74	< 0.0001			
I			0.024	0.081	0.336
II			0.052	0.195	0.591
III			0.069	0.176	0.356

### Non-TC CAUSES of death within 1 year after TC diagnosis

**Fig. 4:**
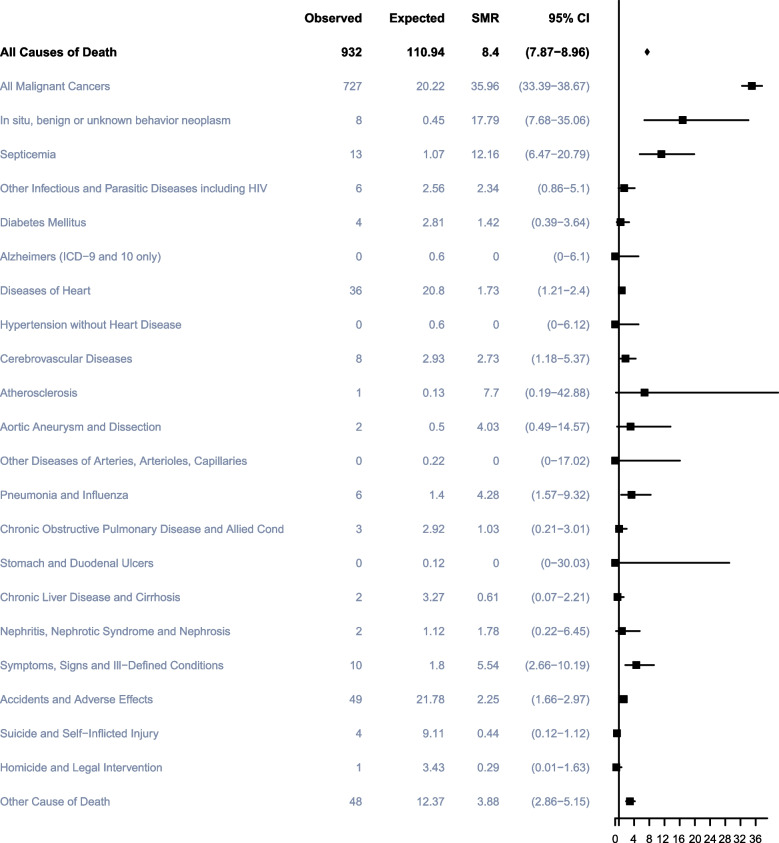
Cause of death of patients within 1 year after diagnosis with testicular cancer. Notes: SMR: Standardized Mortality Ratio

In total, 1,115 TC participants died within the first year after cancer diagnosis, of whom 653 (58.57%) died from TC, 227 (20.36%) died from other cancers, and 235 (21.08%) died from noncancer causes. The most common noncancer cause of death in this period was accidents and adverse effects (53; 4.75%), followed by diseases of heart (45; 4.04%), infectious diseases (27; 2.42%) and vascular diseases (14; 1.26%) (Fig. 2). Men with TC had a statistically significant higher risk of death from all malignant cancers with SMRs of 35.96 (95%CI, 33.39–38.67) within the first year after cancer diagnosis compared with US general population as well as septicemia with SMRs of 12.16 (95%CI, 6.47–20.79), respectively (Fig. 4).

Overall participants and all subgroups except non-Hispanic Black had higher risks of death from all malignant tumors within the first year after TC diagnosis and the non-Hispanic Black had a significantly higher risk of death of atherosclerosis with SMR of 187.85 (95%CI, 4.76–1,046.61). In the first years, the most common cause of noncancer death in participants younger than 40 years old was accidents and adverse effects, while for those over 40, diseases of heart were the commonest cause.

Men diagnosed with TC aged 20–29 years, 40–49 years and non-Hispanic White participants with TC seemed to have a higher risk of dying from diseases of heart within the first year after cancer diagnosis compared with the age- and race-matched general population with SMRs of 5.12 (95%CI, 1.06–14.97), 2.65 (95%CI, 1.37–4.63) and 1.63(95%CI, 1.1–2.31), respectively. Noteworthy, men older than 29 years of age, non-Hispanic White and other ethnic groups had a significantly higher risk of death from septicemia in this year after TC diagnosis, whereas non-Hispanic Black participants had a significantly higher risk of death from Pneumonia and Influenza with SMRs of 27.78 (95%CI, 3.36–100.36) participants aged 40–49 and the non-Hispanic White had higher risks of cerebrovascular disease than that of the general population of US in the first year after cancer diagnosis (SMR, 5.51; 95%CI, 1.14–16.09; SMR, 2.71; 95%CI, 1.09–5.58). However, throughout the follow-up period, non-Hispanic White participants had a lower risk of cerebrovascular disease than the general US population (SMR, 0.68; 95% CI, 0.45–0.99).

### Non-TC Causes of death within 1 to 5 years after TC diagnosis

**Fig. 5:**
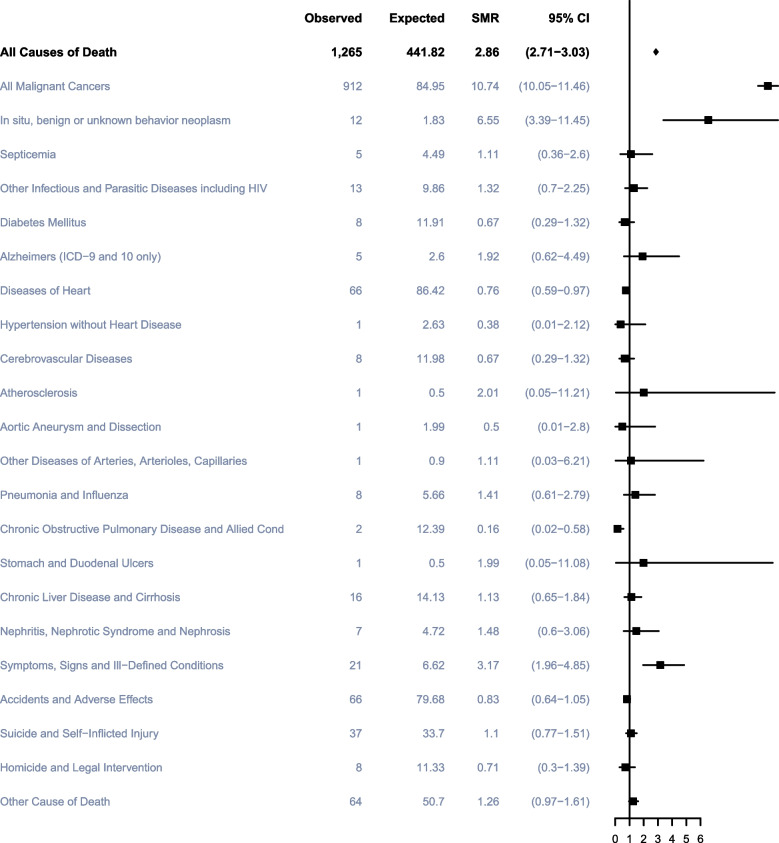
Cause of death of patients within 1 to 5 years after diagnosis with testicular cancer. Notes: SMR: Standardized Mortality Ratio

A total of 1,265 TC participants died within 1 to 5 years of cancer diagnosis, of which 679 (49.74%) died of TC, 304 (22.27%) died of other cancers, and 382 (27.99%) died of non-cancerous causes. The most common noncancer cause of death during the period was also diseases of the heart (75; 5.49%), followed by accidents and adverse effects (73; 5.35%), suicide and self-inflicted injury (37; 2.71%) and infectious diseases (27; 1.98%)(Fig. 2). Men with TC had a statistically significant higher risk of death from all malignant cancers with SMRs of 10.74 (95%CI, 10.05–11.46) within 1 to 5 years after cancer diagnosis compared with US general population as well as symptoms, signs and ill-defined conditions with SMRs of 3.17 (95%CI, 1.96–4.85), respectively. Conversely, participants had a lower risk of dying from diseases of heart and COPD than general population with SMRs of 0.76 (95%CI, 0.59–0.97) and 0.16 (95%CI, 0.02–0.58) in this period of time after TC diagnosis (Fig. 5).

The leading cause of death within 1 to 5 years after TC diagnosis in specific subgroups according to age- and race-related characteristics generally was same as it in the overall population, the risk of dying from all malignant cancer were much higher than that of other causes of deaths.

Compared to the general population, non-Hispanic Black and White and aged 30 to 39 years participants had a significantly higher risk of dying from in situ, benign, or unknown behavior tumors within 1 to 5 years of cancer diagnosis, with SMRs of 32.63, 5.81, and 13.63, respectively. And men aged 30–39 also had a significantly higher risk of death from other infectious and parasitic diseases including HIV as well as Cerebrovascular Diseases with SMRs of 3.06 (95%CI; 1.12–6.65) and 3.88 (95%CI; 1.06–9.94), respectively. Interestingly, participants above the age of 50 have a lower risk of dying from heart disease, diabetes and COPD within 1–5 years of diagnosis. Non-Hispanic White participants had a lower SMR (10.17, 95% CI, 9.48–10.9) of all malignant cancers compared with non-Hispanic Black (12.21, 95% CI, 8.8–16.5) and other races (28.36, 95% CI, 21.84–36.21) in 1 to 5 years after TC diagnosis. In addition, the younger men diagnosed with TC, the higher risk they have of dying from the malignancy within 1–5 years after the diagnosis of TC, compared to the US general population.

### Non-TC Causes of death within 5 to 10 years after TC diagnosis

**Fig. 6:**
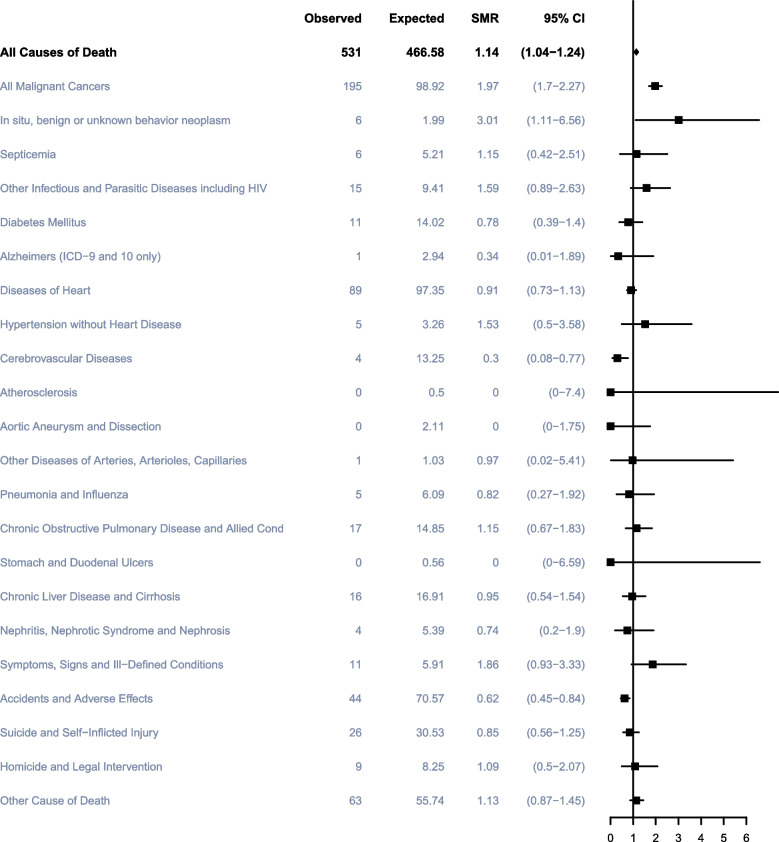
Cause of death of patients within 5 to 10 years after diagnosis with testicular cancer. Notes: SMR: Standardized Mortality Ratio

There were 578 TC men in total who died within 5 to 10 years of diagnosis, including 75 (12.98%) from TC, 145 (25.09%) from other cancers, and 358 (61.94%) from non-cancerous causes. The most common noncancer cause of death during the period was diseases of heart (95; 16.44%), followed by accidents and adverse effects (46; 7.96%), infectious diseases (26; 4.50%), suicide and self-inflicted injury (26; 4.50%) as well as COPD (21; 3.63%) (Fig. 2). Within 5 to 10 years of diagnosis, individuals had a slightly higher overall cause of death than the general male population, with an SMR of 1.14 (95%CI, 1.04–1.24). Among TC men, the risk of death from accidents and adverse effects and cerebrovascular diseases in 5 to 10 years after cancer diagnosis was lower than that in the general male population, with SMRs of 0.62 (95%CI, 0.45–0.84) and 0.30 (95%CI, 0.08–0.77), respectively (Fig. 6).

Interestingly, there were no significant differences in the risk of death from heart diseases in the overall or all subgroups, according to demographic-related characteristics compared with the general population within 5 to 10 years after TC diagnosis. Compared with 5 years after TC diagnosis, the risks of death from all malignant cancers decreased within 5 to 10 years after cancer diagnosis in all subgroups, and there were no significant differences in the risk of death of all malignant tumors in participants who were diagnosed over 50 years, non-Hispanic Black and other race.

### Non-TC Causes of death > 10 years after TC diagnosis

**Fig. 7:**
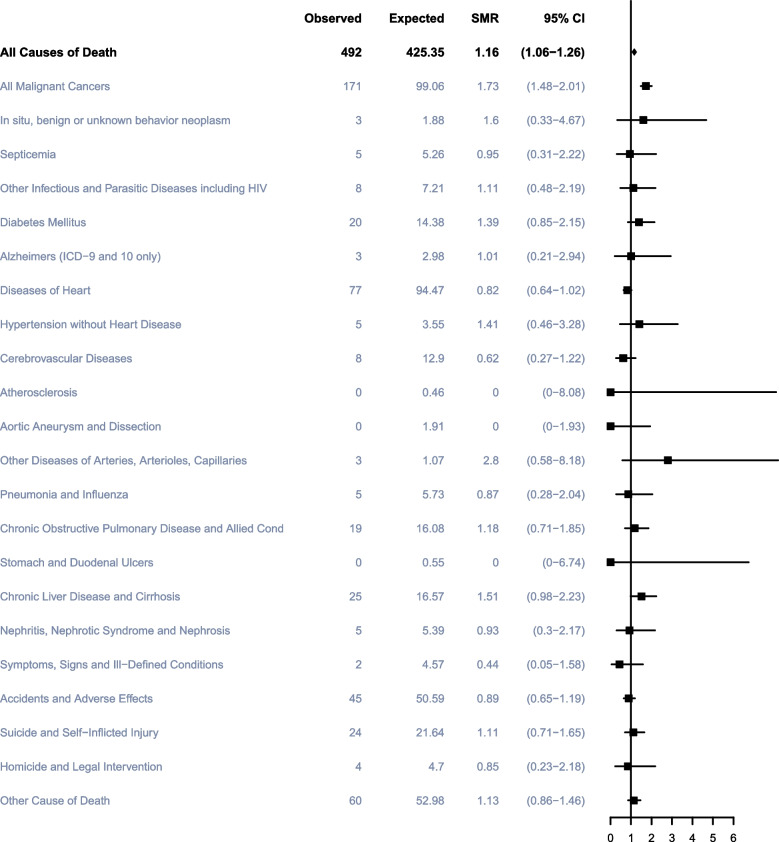
Cause of death of patients more than 10 years after diagnosis with testicular cancer. Notes: SMR: Standardized Mortality Ratio

To date, a total of 515 TC patients passed away 10 years after diagnosis, of whom 38 (7.38%) died from TC, 148 (28.74%) died from other cancers, and 329 (63.88%) died from noncancer causes. The most common noncancer cause was heart diseases (84; 16.31%), followed by accidents and adverse effects (45; 8.74%) and suicide and self-Inflicted Injury (24; 4.66%) (Fig. 2). Over 10 years after TC diagnosis, participants had a slightly higher risk of all-cause mortality with SMRs of 1.16 (95%CI, 1.06–1.26) and a slightly higher risk of dying from all malignant cancers with SMRs of 1.73 (95%CI, 1.48–2.01) compared with the general US population. In the population of men diagnosed more than 10 years, there were no significant differences in the risk of death from diseases of heart in overall and all subgroups. Moreover, we found that participants aged 40 to 49 years had a significantly higher risk (16.90, 95%CI, 2.05–61.05) of death from diseases of arteries, arterioles, capillaries and men of other races had a higher risk (8.64, 95%CI, 1.05–31.19) of death of COPD (Fig. 7).

### Assessment of year of diagnosis

We generated SMRs for men diagnosed with testicular cancer stratified by the year of diagnosis (2000–2010 and 2011–2018) to assess the potential impact of the year of diagnosis on any major results previously reported. We found significant differences in SMRs for deaths from other malignant tumors when stratified by diagnosis year groups (*p* < 0.05). However, this significance disappeared among patients who died from testicular cancer and noncancer causes (Supporting Table 10 and 11).

## Discussion

**Fig. 8:**
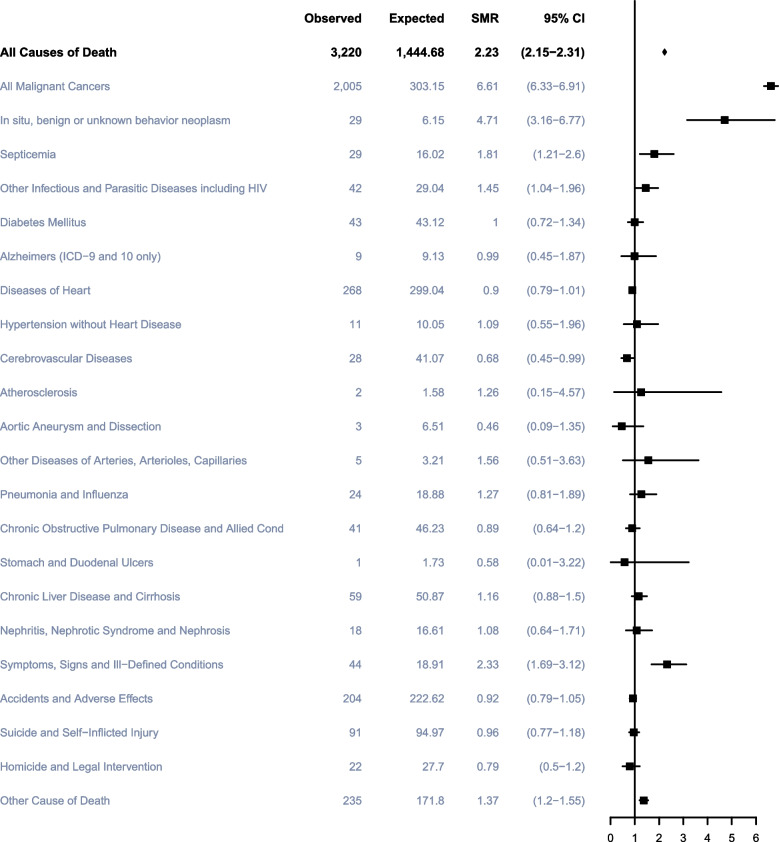
Cause of death for testicular cancer patients. Notes: SMR: Standardized Mortality Ratio

The overall life expectancy and survival rates of patients with TC have significantly improved owing to effective cisplatin-based chemotherapy introduced in the 1970s [17]. Instead, other diseases significantly affected the overall survival of TC patients. Therefore, prevention of death from non-TC causes is crucial when guiding patients about prognosis and life expectancy (Fig. 8). The present study found that the majority of deaths (69.4%) occurred primarily in the first 5 years after diagnosis, and that TC itself was the main cause of death (53.7%) during this period. While beyond 5 years after diagnosis, other cancers became the leading cause of death (26.8%) for TC patients. In addition, the competing risk model has also revealed that different treatment methods significantly affect the competitive events of non-TC deaths among TC patients. Furthermore, tumor staging also has a significant impact on non-TC deaths in TC patients. Generally speaking, the probability of non-TC deaths tends to be higher when the cancer is diagnosed at a later stage. Heart disease was the predominant noncancerous cause of death (16.4%) during this period. Moreover, overall survival analysis of the patient cohort showed that the proportion of non-TC deaths as well as non-cancer deaths increased with a longer latency period after diagnosis. This suggests that clinicians should also focus on screening TC patients for other cancers, as well as heart diseases at different stages of follow-up.

The current study has also discovered that patients with TC have a higher risk of dying from heart diseases within 1 years after diagnosis than that of general population in the United States. In further stratification analysis by different age groups, the older the TC were getting diagnosed, the higher the proportion of death from heart diseases occurred. This finding is in line with the study of Chunkit Fung et al. [10] and they also got a powerful conclusion that increased mortality for heart diseases after chemotherapy was confined to the first year after TC diagnosis and older age at diagnosis was independent risk factors. Besides, in patients diagnosed with TC within 1 years, deaths due to cerebrovascular diseases were associated with significantly higher risks compared with the general population (SMR, 2.73; 95%CI, 1.18–5.37), which was in agreement with the study by Chunkit Fung et al. [10]. And they also reported that cerebrovascular disease mortality with significantly elevated risks after chemotherapy but not surgery alone [10]. The occurrence of cerebrovascular events after cisplatin-based chemotherapy were often reported [18–20], which may be related to higher incidences of acute thromboembolic events (TEE) after cisplatin-based chemotherapy to some extent [21, 22] and relevant studies have been looking for potential predicters of such acute accidents [18, 23].

The morbidity of second cancers (SC) in long-term testicular cancer survivor is much higher than that in the U.S. general population. The morbidity and location of SC in patients with different treatment methods were obviously different. Men with TC treated with surgery had a slightly higher risk of SC, while the overall risk of SC was significantly higher among survivors treated with chemotherapy and radiotherapy [24, 25]. Consistent with that of previous published articles, we found similar results that the proportion of deaths from other cancer occupied the first place in non-TC causes of death at all stages of follow-up and this proportion has exceeded TC itself after 5 years with TC. In addition, a similar trend was observed in a cohort study conducted by Luyao Zhang et al., where the researchers calculated the RR value indicating that TC patients have a higher burden of non-TC and TC death than the general population [26]. Decreasing SC mortality risk with increasing age at TC diagnosis has been confirmed by Ragnhild Hellesnes et al. [27] and the Dutch study [6] and young age at TC diagnosis has been associated with an elevated SC incidence in previous studies [24, 28]. Bladder and kidney cancer were the frequent SCs, which might be related to the long-term remnant and renal clearance of cisplatin. In addition, cancers of the small intestine, lung, and thyroid and soft tissue sarcoma were also common SCs [5, 25, 26, 29, 30].

The incidence of suicidal death has been noted to be higher among cancer patients [31, 32]. However, in our study, the TC population in different race, age groups and follow-up periods after diagnosis did not have an increased suicidal risk after cancer diagnosis. Previous studies have reported an increased risk of sepsis after chemotherapy [33–36], and in our study, we also found a higher risk of death from sepsis and other infectious and parasitic diseases (including HIV) in TC patients.

Nowadays, secondary analysis based on public data and research has provided convenience for researchers [37–40]. However, the current study has some limitations. First, it was a retrospective study conducted by SEER database and thus presented inherent limitations. There may be statistical bias for the true COD in elderly patients with multiple comorbidities. Second, since the SEER database has “yes” and 'no/unknown' information statistics for chemotherapy and lacks specific chemotherapy regimens, this study did not stratify the results for chemotherapy in order to avoid excessive bias. Finally, this study used a U.S. population, so more cohort studies are needed to demonstrate whether the findings can be generalized to a larger scale.

## Conclusions

Previous studies have reported on non-TC mortality rates in testicular cancer. Our study is based on a large sample from the SEER database, with a long-term study period of over 10 years. Part of our research confirms the relevant conclusions of published articles. These findings may provide insight into how men with TC should be counseled regarding future health risks and highlight the importance of multidisciplinary care for such patients.

### Supplementary Information

**Additional file 1:Fig. S1.** Testicular cancer diagnosis trends in different race groups.

**Additional file 2:Fig. S2.** Cumulative incidence curves of cause-specific death according to stage.

**Additional file 3:** **Table S1.** Cause of death for testicular cancer patients <20 years. **Table S2.** Cause of death for testicular cancer patients 20-29 years. **Table S3.** Cause of death for testicular cancer patients 30-39 years. **Table S4.** Cause of death for testicular cancer patients 40-49 years. **Table S5.** Cause of death for testicular cancer patients 50+ years. **Table S6.** Cause of death for testicular cancer in white. **Table S7.** Cause of death for testicular cancer in black. **Table S8.** Cause of death for testicular cancer in other. **Table S9.** Definition of each cause of death and corresponding codes in the ICD-10 of Diseases and Related Health. **Table 10.** Impact of year of diagnosis on different categories of COD. **Table 11.**

## Data Availability

The SEER*Stat software (version 8.3.9) was used to obtain patient information from the SEER database during the current study (https://seer.cancer.gov/data/options.html). A registration form needs to be completed before using and filter criteria need to be added. If reasonable requests are made, the datasets can also be obtained from the corresponding author.

## References

[1] Albers P (2015). Guidelines on testicular cancer: 2015 Update. Eur Urol.

[2] Siegel RL (2021). Cancer statistics, 2021. CA Cancer J Clin.

[3] Trama A (2015). Survival of male genital cancers (prostate, testis and penis) in Europe 1999–2007: Results from the EUROCARE-5 study. Eur J Cancer.

[4] Verhoeven RH (2014). Markedly increased incidence and improved survival of testicular cancer in the Netherlands. Acta Oncol.

[5] Verhoeven RHA (2013). Testicular cancer in Europe and the USA: survival still rising among older patients. Ann Oncol.

[6] Groot HJ (2018). Risk of solid cancer after treatment of testicular germ cell cancer in the platinum Era. J Clin Oncol.

[7] Groot HJ (2020). Platinum exposure and cause-specific mortality among patients with testicular cancer. Cancer.

[8] Kier MG (2017). Prognostic factors and treatment results after bleomycin, etoposide, and cisplatin in germ cell cancer: a population-based study. Eur Urol.

[9] van den Belt-Dusebout AW (2007). Treatment-specific risks of second malignancies and cardiovascular disease in 5-year survivors of testicular cancer. J Clin Oncol.

[10] Schaffar R (2019). Testicular cancer in Geneva, Switzerland, 1970–2012: incidence trends, survival and risk of second cancer. BMC Urol.

[11] Fung C (2015). Cardiovascular disease mortality after chemotherapy or surgery for testicular nonseminoma: a population-based study. J Clin Oncol.

[12] Surveillance Research Program, National Cancer Institute. SEER*Stat software, version 8.3.6. Released August 2019; Available from: www.seer.cancer.gov/seerstat.

[13] Surveillance, Epidemiology, and End Results (SEER) Program. SEER Cause of Death Recode 1969+ (03/01/2018) SEER Data Reporting Tools. https://seer.cancer.gov/codrecode/1969_d03012018/index.html. Available from Cited 1 July 2019.

[14] Fossa SD (2007). Noncancer causes of death in survivors of testicular cancer. J Natl Cancer Inst.

[15] Afifi AM (2020). Causes of death after breast cancer diagnosis: A US population-based analysis. Cancer.

[16] Cederkvist L (2019). Modeling the cumulative incidence function of multivariate competing risks data allowing for within-cluster dependence of risk and timing. Biostatistics.

[17] Austin PC, Fine JP (2017). Practical recommendations for reporting Fine-Gray model analyses for competing risk data. Stat Med.

[18] Blackford AL (2020). Recent Trends in the Incidence and Survival of Stage 1A Pancreatic Cancer: A Surveillance, Epidemiology, and End Results Analysis. J Natl Cancer Inst.

[19] Kim HJ (2000). Permutation tests for joinpoint regression with applications to cancer rates. Stat Med.

[20] Fung C (2019). Testicular Cancer Survivorship. J Natl Compr Canc Netw.

[21] Einhorn LH, Donohue J (1977). Cis-diamminedichloroplatinum, vinblastine, and bleomycin combination chemotherapy in disseminated testicular cancer. Ann Intern Med.

[22] O'Reilly A (2014). Testicular cancer and platinum: a double-edged sword. J Clin Oncol.

[23] Azak A (2008). Cerebrovascular accident during cisplatin-based combination chemotherapy of testicular germ cell tumor: an unusual case report. Anticancer Drugs.

[24] Piketty AC (2005). The risk of thrombo-embolic events is increased in patients with germ-cell tumours and can be predicted by serum lactate dehydrogenase and body surface area. Br J Cancer.

[25] van den Belt-Dusebout AW (2006). Long-term risk of cardiovascular disease in 5-year survivors of testicular cancer. J Clin Oncol.

[26] Kier MG (2016). Second malignant neoplasms and cause of death in patients with germ cell cancer: a danish nationwide cohort study. JAMA Oncol.

[27] Gizzi M (2016). Predicting and preventing thromboembolic events in patients receiving cisplatin-based chemotherapy for germ cell tumours. Eur J Cancer.

[28] Hellesnes R (2020). Continuing increased risk of second cancer in long-term testicular cancer survivors after treatment in the cisplatin era. Int J Cancer.

[29] Zhang L (2019). Second cancers and causes of death in patients with testicular cancer in Sweden. PLoS ONE.

[30] Hellesnes R (2021). Testicular cancer in the cisplatin era: causes of death and mortality rates in a population-based cohort. J Clin Oncol.

[31] Travis LB (2005). Second cancers among 40,576 testicular cancer patients: focus on long-term survivors. J Natl Cancer Inst.

[32] Fung C (2013). Solid tumors after chemotherapy or surgery for testicular nonseminoma: a population-based study. J Clin Oncol.

[33] Hjelle LV (2015). Long-term platinum retention after platinum-based chemotherapy in testicular cancer survivors: a 20-year follow-up study. Anticancer Res.

[34] Gerl A, Schierl R (2000). Urinary excretion of platinum in chemotherapy-treated long-term survivors of testicular cancer. Acta Oncol.

[35] Saad AM (2019). Suicidal death within a year of a cancer diagnosis: A population-based study. Cancer.

[36] Lorch A (2007). Single versus sequential high-dose chemotherapy in patients with relapsed or refractory germ cell tumors: a prospective randomized multicenter trial of the German Testicular Cancer Study Group. J Clin Oncol.

[37] Teoh EM (2010). The efficacy of preventing neutropenic sepsis in patients with testicular germ cell tumours: results of two consecutive audits. Clin Oncol (R Coll Radiol).

[38] Zhang X (2021). Pyrethroids Toxicity to Male Reproductive System and Offspring as a Function of Oxidative Stress Induction: Rodent Studies. Front Endocrinol (Lausanne).

[39] Zhang T (2022). Gut microbiota may contribute to the postnatal male reproductive abnormalities induced by prenatal dibutyl phthalate exposure. Chemosphere.

[40] Yu L (2021). Multi-omics analysis reveals the interaction between the complement system and the coagulation cascade in the development of endometriosis. Sci Rep.

