# Supplementary material for: Causes of death after testicular cancer diagnosis: a US population-based analysis
Source: BMC Urol. 2023 Sep 2;23:144. doi: 10.1186/s12894-023-01309-3 (PMC10475185; doi:10.1186/s12894-023-01309-3)
Supplement: Supplementary file 1 — Additional file 1:Fig. S1. Testicular cancer diagnosis trends in different race groups. [file 12894_2023_1309_MOESM1_ESM.pdf]

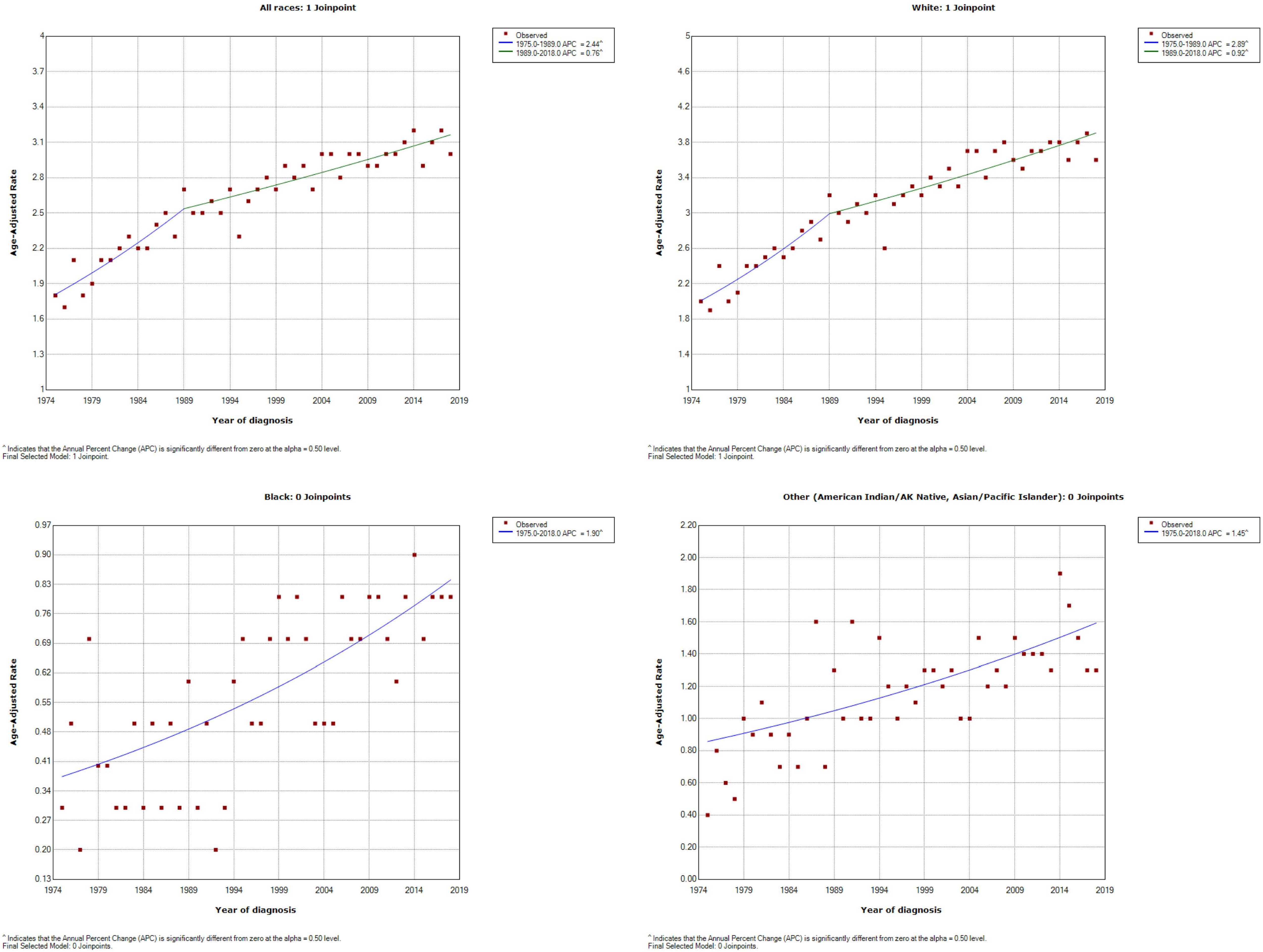

Supporting figure 1. Testicular cancer diagnosis trends in different race groups.
