# Supplementary figures and images for: Causes of death after testicular cancer diagnosis: a US population-based analysis
Source: BMC Urol. 2023 Sep 2;23:144. doi: 10.1186/s12894-023-01309-3 (PMC10475185; doi:10.1186/s12894-023-01309-3)

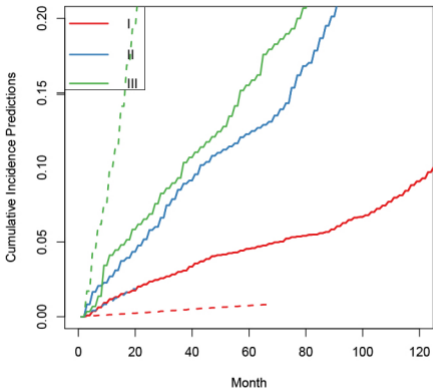

Supporting figure 2. Cumulative incidence curves of cause-specific death according to Stage.

Supplement: Supplementary file 2 — Additional file 2:Fig. S2. Cumulative incidence curves of cause-specific death according to stage. [file 12894_2023_1309_MOESM2_ESM.pdf]
