# Supplementary material for: Causes of death after testicular cancer diagnosis: a US population-based analysis
Source: BMC Urol. 2023 Sep 2;23:144. doi: 10.1186/s12894-023-01309-3 (PMC10475185; doi:10.1186/s12894-023-01309-3)
Supplement: Supplementary file 3 — Additional file 3: Table S1. Cause of death for testicular cancer patients <20 years. Table S2. Cause of death for testicular cancer patients 20-29 years. Table S3. Cause of death for testicular cancer patients 30-39 years. Table S4. Cause of death for testicular cancer patients 40-49 years. Table S5. Cause of death for testicular cancer patients 50+ years. Table S6. Cause of death for testicular cancer in white. Table S7. Cause of death for testicular cancer in black. Table S8. Cause of death for testicular cancer in other. Table S9. Definition of each cause of death and corresponding codes in the ICD-10 of Diseases and Related Health. Table 10. Impact of year of diagnosis on different categories of COD. Table 11. [file 12894_2023_1309_MOESM3_ESM.docx]

| Supporting Table 1. Cause of death for testicular cancer patients <20 years. | | | | | | | | | | |  |
| --- | --- | --- | --- | --- | --- | --- | --- | --- | --- | --- | --- |
|  | Total | | <1 years | | 1-5 years | | 5-10 years | | >10 years | | |
| Selected Events | Observed^1^ | SMR^2^  (95% CI^3^) | Observed | SMR | Observed | SMR | Observed | SMR | Observed | SMR | |
| All Causes of Death | 68 | 16.83^#^(13.07-21.33) | 35 | 24.08^#^(16.77-33.49) | 32 | 14.27^#^(9.76-20.15) | 1 | 5.02(0.13-27.97) | 0 | 0 | |
| All Malignant Cancers | 60 | 311.41^#^(237.64-400.85) | 30 | 469.85^#^(317.01-670.74) | 29 | 294.27^#^(197.07-422.62) | 1 | 50.53^#^(1.28-281.56) | 0 | 0 | |
| In situ, benign or unknown behavior neoplasm | 1 | 70.92^#^(1.8-395.16) | 0 | 0(0-826.1) | 1 | 132.68^#^(3.36-739.22) | 0 | 0(0-2566.16) | 0 | 0 | |
| Septicemia | 0 | 0(0-186.61) | 0 | 0(0-571.01) | 0 | 0(0-327) | 0 | 0(0-2651.21) | 0 | 0 | |
| Other Infectious and Parasitic Diseases including HIV | 0 | 0(0-158.71) | 0 | 0(0-471.46) | 0 | 0(0-280.69) | 0 | 0(0-2301.08) | 0 | 0 | |
| Diabetes Mellitus | 0 | 0(0-342.52) | 0 | 0(0-972.28) | 0 | 0(0-674.87) | 0 | 0(0-4521.17) | 0 | 0 | |
| Alzheimers | 0 | 0(0-0) | 0 | 0(0-0) | 0 | 0(0-0) | 0 | 0(0-0) | 0 | 0 | |
| Diseases of Heart | 0 | 0(0-39.57) | 0 | 0(0-111.57) | 0 | 0(0-73.26) | 0 | 0(0-630.94) | 0 | 0 | |
| Hypertension without Heart Disease | 0 | 0(0-5282.46) | 0 | 0(0-14599.05) | 0 | 0(0-9808.94) | 0 | 0(0-75802.13) | 0 | 0 | |
| Cerebrovascular Diseases | 0 | 0(0-171.45) | 0 | 0(0-525.84) | 0 | 0(0-319.36) | 0 | 0(0-1928.37) | 0 | 0 | |
| Atherosclerosis | 0 | 0(0-18531.04) | 0 | 0(0-63226.59) | 0 | 0(0-30041.29) | 0 | 0(0-291159.43) | 0 | 0 | |
| Aortic Aneurysm and Dissection | 0 | 0(0-1004.93) | 0 | 0(0-2658.19) | 0 | 0(0-1933.09) | 0 | 0(0-18872.08) | 0 | 0 | |
| Other Diseases of Arteries, Arterioles, Capillaries | 0 | 0(0-1850.94) | 0 | 0(0-5300.3) | 0 | 0(0-3388.66) | 0 | 0(0-28932.27) | 0 | 0 | |
| Pneumonia and Influenza | 0 | 0(0-126.68) | 0 | 0(0-385.53) | 0 | 0(0-224.62) | 0 | 0(0-1743.1) | 0 | 0 | |
| Chronic Obstructive Pulmonary Disease and Allied Cond | 0 | 0(0-197.36) | 0 | 0(0-679.09) | 0 | 0(0-390.28) | 0 | 0(0-1349.02) | 0 | 0 | |
| Stomach and Duodenal Ulcers | 0 | 0(0-7211.13) | 0 | 0(0-20147.99) | 0 | 0(0-13994.54) | 0 | 0(0-86866.12) | 0 | 0 | |
| Chronic Liver Disease and Cirrhosis | 0 | 0(0-4856.31) | 0 | 0(0-13503.78) | 0 | 0(0-8830.32) | 0 | 0(0-90352.68) | 0 | 0 | |
| Nephritis, Nephrotic Syndrome and Nephrosis | 0 | 0(0-468.04) | 0 | 0(0-1360.63) | 0 | 0(0-796.86) | 0 | 0(0-10224.11) | 0 | 0 | |
| Symptoms, Signs and Ill-Defined Conditions | 0 | 0(0-22.27) | 0 | 0(0-64.84) | 0 | 0(0-35.73) | 0 | 0(0-1103.96) | 0 | 0 | |
| Accidents and Adverse Effects | 3 | 2(0.41-5.85) | 2 | 3.5(0.42-12.65) | 1 | 1.25(0.03-6.96) | 0 | 0(0-51.39) | 0 | 0 | |
| Suicide and Self-Inflicted Injury | 0 | 0(0-6.43) | 0 | 0(0-17.14) | 0 | 0(0-12.31) | 0 | 0(0-144.12) | 0 | 0 | |
| Homicide and Legal Intervention | 1 | 2.72(0.07-15.17) | 0 | 0(0-27.89) | 1 | 5.29(0.13-29.5) | 0 | 0(0-132.76) | 0 | 0 | |
| Other Cause of Death | 3 | 8.83^#^(1.82-25.82) | 3 | 26.02^#^(5.37-76.05) | 0 | 0(0-19.89) | 0 | 0(0-150.14) | 0 | 0 | |

**1** number of cancer patients who died due to each cause of death

**2** standardized mortality rate

**3** 95% Confidence interval

**^#^** P value less than .05

| Supporting Table 2. Cause of death for testicular cancer patients 20-29 years. | | | | | | | | | | |  |
| --- | --- | --- | --- | --- | --- | --- | --- | --- | --- | --- | --- |
|  | Total | | <1 years | | 1-5 years | | 5-10 years | | >10 years | | |
| Selected Events | Observed^1^ | SMR^2^  (95% CI^3^) | Observed | SMR | Observed | SMR | Observed | SMR | Observed | SMR | |
| All Causes of Death | 621 | 8.04^#^(7.42-8.7) | 236 | 15.95^#^(13.98-18.12) | 340 | 8.05^#^(7.21-8.95) | 41 | 2.28^#^(1.63-3.09) | 4 | 1.83(0.5-4.68) | |
| All Malignant Cancers | 510 | 143.07^#^(130.92-156.05) | 200 | 292.01^#^(252.94-335.4) | 291 | 148.62^#^(132.03-166.71) | 17 | 20.59^#^(11.99-32.96) | 2 | 20.87^#^(2.53-75.38) | |
| In situ, benign or unknown behavior neoplasm | 8 | 46.50^#^(20.08-91.63) | 2 | 60.14^#^(7.28-217.26) | 4 | 42.37^#^(11.54-108.49) | 2 | 50.28^#^(6.09-181.64) | 0 | 0(0-802.13) | |
| Septicemia | 2 | 6.56(0.79-23.68) | 1 | 17.89(0.45-99.65) | 1 | 6.05(0.15-33.72) | 0 | 0(0-49.37) | 0 | 0(0-400.8) | |
| Other Infectious and Parasitic Diseases including HIV | 3 | 4.54(0.94-13.26) | 2 | 15.26^#^(1.85-55.14) | 0 | 0(0-10.11) | 1 | 6.77(0.17-37.7) | 0 | 0(0-211.1) | |
| Diabetes Mellitus | 1 | 1.82(0.05-10.13) | 0 | 0(0-36.96) | 1 | 3.39(0.09-18.87) | 0 | 0(0-26.94) | 0 | 0(0-204.26) | |
| Alzheimers | 1 | 1,500.42^#^(37.99-8359.8) | 0 | 0(0-30579.81) | 1 | 2,766.21^#^(70.03-15412.34) | 0 | 0(0-22512.68) | 0 | 0(0-180066.53) | |
| Diseases of Heart | 5 | 1.58(0.51-3.68) | 3 | 5.12^#^(1.06-14.97) | 2 | 1.16(0.14-4.2) | 0 | 0(0-4.79) | 0 | 0(0-39.1) | |
| Hypertension without Heart Disease | 0 | 0(0-57.41) | 0 | 0(0-317.09) | 0 | 0(0-107.11) | 0 | 0(0-229.48) | 0 | 0(0-1752.33) | |
| Cerebrovascular Diseases | 1 | 2.23(0.06-12.43) | 1 | 11.75(0.3-65.44) | 0 | 0(0-15.11) | 0 | 0(0-34.85) | 0 | 0(0-283.26) | |
| Atherosclerosis | 0 | 0(0-706.71) | 0 | 0(0-3629) | 0 | 0(0-1282.44) | 0 | 0(0-3078.66) | 0 | 0(0-28680.5) | |
| Aortic Aneurysm and Dissection | 0 | 0(0-27.76) | 0 | 0(0-147.73) | 0 | 0(0-51.16) | 0 | 0(0-115.83) | 0 | 0(0-927.12) | |
| Other Diseases of Arteries, Arterioles, Capillaries | 0 | 0(0-90.86) | 0 | 0(0-485.56) | 0 | 0(0-168.57) | 0 | 0(0-375.39) | 0 | 0(0-2855.77) | |
| Pneumonia and Influenza | 1 | 2.1(0.05-11.69) | 0 | 0(0-41.33) | 1 | 3.83(0.1-21.34) | 0 | 0(0-32.58) | 0 | 0(0-283.97) | |
| Chronic Obstructive Pulmonary Disease and Allied Cond | 0 | 0(0-13.02) | 0 | 0(0-68.92) | 0 | 0(0-23.98) | 0 | 0(0-54.72) | 0 | 0(0-435.27) | |
| Stomach and Duodenal Ulcers | 0 | 0(0-169.45) | 0 | 0(0-902.43) | 0 | 0(0-308.4) | 0 | 0(0-718.43) | 0 | 0(0-6290.01) | |
| Chronic Liver Disease and Cirrhosis | 0 | 0(0-9.28) | 0 | 0(0-57.54) | 0 | 0(0-17.95) | 0 | 0(0-33.09) | 0 | 0(0-224.21) | |
| Nephritis, Nephrotic Syndrome and Nephrosis | 0 | 0(0-20.76) | 0 | 0(0-109.08) | 0 | 0(0-37.98) | 0 | 0(0-88.41) | 0 | 0(0-730.38) | |
| Symptoms, Signs and Ill-Defined Conditions | 10 | 5.89^#^(2.83-10.84) | 5 | 14.76^#^(4.79-34.45) | 3 | 3.2(0.66-9.34) | 2 | 5.32(0.64-19.2) | 0 | 0(0-84.69) | |
| Accidents and Adverse Effects | 39 | 1.06(0.75-1.45) | 11 | 1.57(0.78-2.81) | 15 | 0.75(0.42-1.23) | 11 | 1.28(0.64-2.29) | 2 | 1.91(0.23-6.89) | |
| Suicide and Self-Inflicted Injury | 16 | 1.14(0.65-1.85) | 1 | 0.37(0.01-2.08) | 13 | 1.69(0.9-2.89) | 2 | 0.61(0.07-2.19) | 0 | 0(0-9.13) | |
| Homicide and Legal Intervention | 4 | 0.57(0.16-1.46) | 1 | 0.7(0.02-3.92) | 2 | 0.52(0.06-1.86) | 1 | 0.66(0.02-3.66) | 0 | 0(0-20.06) | |
| Other Cause of Death | 16 | 2.43^#^(1.39-3.95) | 8 | 6.43^#^(2.78-12.68) | 4 | 1.12(0.3-2.86) | 4 | 2.57(0.7-6.59) | 0 | 0(0-19.44) | |

**1** number of cancer patients who died due to each cause of death

**2** standardized mortality rate

**3** 95% Confidence interval

**^#^** P value less than .05

| Supporting Table 3. Cause of death for testicular cancer patients 30-39 years. | | | | | | | | | | |  |
| --- | --- | --- | --- | --- | --- | --- | --- | --- | --- | --- | --- |
|  | Total | | <1 years | | 1-5 years | | 5-10 years | | >10 years | | |
| Selected Events | Observed^1^ | SMR^2^  (95% CI^3^) | Observed | SMR | Observed | SMR | Observed | SMR | Observed | SMR | |
| All Causes of Death | 678 | 3.59^#^(3.33-3.87) | 215 | 10.72^#^(9.33-12.25) | 323 | 4.29^#^(3.83-4.78) | 93 | 1.47^#^(1.19-1.81) | 47 | 1.56^#^(1.14-2.07) | |
| All Malignant Cancers | 454 | 30.82^#^(28.05-33.79) | 163 | 101.06^#^(86.14-117.82) | 232 | 38.84^#^(34-44.17) | 38 | 7.76^#^(5.49-10.65) | 21 | 9.34^#^(5.78-14.27) | |
| In situ, benign or unknown behavior neoplasm | 7 | 13.33^#^(5.36-27.46) | 2 | 33.12^#^(4.01-119.64) | 3 | 13.63^#^(2.81-39.84) | 1 | 5.81(0.15-32.36) | 1 | 13.78(0.35-76.75) | |
| Septicemia | 5 | 3.97^#^(1.29-9.27) | 4 | 30.41^#^(8.29-77.87) | 0 | 0(0-7.42) | 0 | 0(0-8.68) | 1 | 4.88(0.12-27.19) | |
| Other Infectious and Parasitic Diseases including HIV | 12 | 2.87^#^(1.48-5.01) | 3 | 4.82(0.99-14.1) | 6 | 3.06^#^(1.12-6.65) | 3 | 2.54(0.52-7.41) | 0 | 0(0-8.86) | |
| Diabetes Mellitus | 6 | 1.85(0.68-4.04) | 0 | 0(0-10.86) | 2 | 1.56(0.19-5.64) | 2 | 1.84(0.22-6.64) | 2 | 3.79(0.46-13.71) | |
| Alzheimers | 0 | 0(0-524.82) | 0 | 0(0-4661.8) | 0 | 0(0-1333.43) | 0 | 0(0-1596.85) | 0 | 0(0-3177.21) | |
| Diseases of Heart | 17 | 0.89(0.52-1.42) | 3 | 1.46(0.3-4.26) | 7 | 0.91(0.37-1.87) | 6 | 0.94(0.34-2.04) | 1 | 0.34(0.01-1.88) | |
| Hypertension without Heart Disease | 0 | 0(0-7.04) | 0 | 0(0-71.58) | 0 | 0(0-18.45) | 0 | 0(0-20.58) | 0 | 0(0-39.58) | |
| Cerebrovascular Diseases | 5 | 1.95(0.63-4.55) | 1 | 3.63(0.09-20.22) | 4 | 3.88^#^(1.06-9.94) | 0 | 0(0-4.31) | 0 | 0(0-9.13) | |
| Atherosclerosis | 0 | 0(0-107.31) | 0 | 0(0-895.53) | 0 | 0(0-257.08) | 0 | 0(0-338.33) | 0 | 0(0-737.36) | |
| Aortic Aneurysm and Dissection | 0 | 0(0-5.13) | 0 | 0(0-48.96) | 0 | 0(0-12.86) | 0 | 0(0-15.16) | 0 | 0(0-32.39) | |
| Other Diseases of Arteries, Arterioles, Capillaries | 0 | 0(0-21.21) | 0 | 0(0-208.35) | 0 | 0(0-54.88) | 0 | 0(0-63.23) | 0 | 0(0-120.31) | |
| Pneumonia and Influenza | 4 | 2.47(0.67-6.32) | 2 | 11.60^#^(1.41-41.92) | 2 | 3.07(0.37-11.08) | 0 | 0(0-6.74) | 0 | 0(0-14.82) | |
| Chronic Obstructive Pulmonary Disease and Allied Cond | 0 | 0(0-4.05) | 0 | 0(0-37.39) | 0 | 0(0-10.13) | 0 | 0(0-12.15) | 0 | 0(0-25.33) | |
| Stomach and Duodenal Ulcers | 0 | 0(0-29.16) | 0 | 0(0-270.79) | 0 | 0(0-72.75) | 0 | 0(0-88.09) | 0 | 0(0-181.79) | |
| Chronic Liver Disease and Cirrhosis | 11 | 2.23^#^(1.12-4) | 2 | 4.08(0.49-14.75) | 4 | 2.14(0.58-5.47) | 4 | 2.39(0.65-6.13) | 1 | 1.12(0.03-6.25) | |
| Nephritis, Nephrotic Syndrome and Nephrosis | 0 | 0(0-3.71) | 0 | 0(0-35.29) | 0 | 0(0-9.27) | 0 | 0(0-11) | 0 | 0(0-23.66) | |
| Symptoms, Signs and Ill-Defined Conditions | 13 | 3.25^#^(1.73-5.56) | 1 | 2.07(0.05-11.55) | 10 | 5.90^#^(2.83-10.85) | 2 | 1.58(0.19-5.72) | 0 | 0(0-6.61) | |
| Accidents and Adverse Effects | 60 | 0.87(0.66-1.12) | 22 | 3.13^#^(1.96-4.74) | 20 | 0.74(0.45-1.14) | 10 | 0.43^#^(0.2-0.79) | 8 | 0.68(0.3-1.35) | |
| Suicide and Self-Inflicted Injury | 28 | 1(0.66-1.44) | 1 | 0.34(0.01-1.89) | 12 | 1.07(0.55-1.87) | 10 | 1.05(0.51-1.94) | 5 | 1.12(0.36-2.62) | |
| Homicide and Legal Intervention | 7 | 0.68(0.27-1.41) | 0 | 0(0-3.16) | 3 | 0.71(0.15-2.06) | 3 | 0.9(0.19-2.63) | 1 | 0.67(0.02-3.75) | |
| Other Cause of Death | 49 | 2.42^#^(1.79-3.2) | 11 | 5.10^#^(2.55-9.13) | 18 | 2.23^#^(1.32-3.52) | 14 | 2.07^#^(1.13-3.47) | 6 | 1.83(0.67-3.99) | |

**1** number of cancer patients who died due to each cause of death

**2** standardized mortality rate

**3** 95% Confidence interval

**^#^** P value less than .05

| Supporting Table 4. Cause of death for testicular cancer patients 40-49 years. | | | | | | | | | | |  |
| --- | --- | --- | --- | --- | --- | --- | --- | --- | --- | --- | --- |
|  | Total | | <1 years | | 1-5 years | | 5-10 years | | >10 years | | |
| Selected Events | Observed^1^ | SMR^2^  (95% CI^3^) | Observed | SMR | Observed | SMR | Observed | SMR | Observed | SMR | |
| All Causes of Death | 660 | 2.20^#^(2.04-2.38) | 180 | 7.88^#^(6.77-9.12) | 261 | 2.71^#^(2.39-3.06) | 119 | 1.16(0.96-1.38) | 100 | 1.29^#^(1.05-1.57) | |
| All Malignant Cancers | 387 | 8.40^#^(7.59-9.28) | 135 | 37.04^#^(31.06-43.85) | 172 | 11.35^#^(9.72-13.18) | 46 | 2.90^#^(2.12-3.87) | 34 | 2.99^#^(2.07-4.18) | |
| In situ, benign or unknown behavior neoplasm | 2 | 2.17(0.26-7.83) | 1 | 13.49(0.34-75.19) | 1 | 3.25(0.08-18.12) | 0 | 0(0-11.59) | 0 | 0(0-16.51) | |
| Septicemia | 7 | 2.41(0.97-4.96) | 3 | 14.16^#^(2.92-41.39) | 2 | 2.2(0.27-7.95) | 1 | 1(0.03-5.54) | 1 | 1.27(0.03-7.1) | |
| Other Infectious and Parasitic Diseases including HIV | 12 | 1.28(0.66-2.24) | 1 | 1.07(0.03-5.98) | 4 | 1.11(0.3-2.84) | 4 | 1.29(0.35-3.3) | 3 | 1.75(0.36-5.11) | |
| Diabetes Mellitus | 10 | 1.14(0.55-2.09) | 1 | 1.58(0.04-8.83) | 3 | 1.11(0.23-3.24) | 3 | 0.99(0.2-2.9) | 3 | 1.24(0.25-3.61) | |
| Alzheimers | 0 | 0(0-58.37) | 0 | 0(0-808.65) | 0 | 0(0-181.74) | 0 | 0(0-164.17) | 0 | 0(0-232.47) | |
| Diseases of Heart | 54 | 0.93(0.7-1.21) | 12 | 2.65^#^(1.37-4.63) | 17 | 0.9(0.52-1.44) | 14 | 0.7(0.38-1.17) | 11 | 0.75(0.37-1.34) | |
| Hypertension without Heart Disease | 0 | 0(0-2.11) | 0 | 0(0-31.22) | 0 | 0(0-7.08) | 0 | 0(0-6.09) | 0 | 0(0-7.28) | |
| Cerebrovascular Diseases | 4 | 0.57(0.15-1.45) | 3 | 5.51^#^(1.14-16.09) | 0 | 0(0-1.62) | 0 | 0(0-1.52) | 1 | 0.55(0.01-3.07) | |
| Atherosclerosis | 0 | 0(0-24.1) | 0 | 0(0-268.99) | 0 | 0(0-68.65) | 0 | 0(0-71.25) | 0 | 0(0-109.09) | |
| Aortic Aneurysm and Dissection | 2 | 1.39(0.17-5.02) | 1 | 9.3(0.24-51.84) | 1 | 2.19(0.06-12.2) | 0 | 0(0-7.45) | 0 | 0(0-9.73) | |
| Other Diseases of Arteries, Arterioles, Capillaries | 2 | 4.61(0.56-16.67) | 0 | 0(0-114.01) | 0 | 0(0-27.22) | 0 | 0(0-25.04) | 2 | 16.90^#^(2.05-61.05) | |
| Pneumonia and Influenza | 5 | 1.58(0.51-3.69) | 1 | 4.19(0.11-23.33) | 3 | 2.96(0.61-8.64) | 0 | 0(0-3.37) | 1 | 1.23(0.03-6.87) | |
| Chronic Obstructive Pulmonary Disease and Allied Cond | 2 | 0.61(0.07-2.19) | 0 | 0(0-14.66) | 0 | 0(0-3.46) | 2 | 1.74(0.21-6.3) | 0 | 0(0-4.42) | |
| Stomach and Duodenal Ulcers | 1 | 2.66(0.07-14.79) | 0 | 0(0-122.96) | 1 | 8.09(0.2-45.08) | 0 | 0(0-28.77) | 0 | 0(0-38.9) | |
| Chronic Liver Disease and Cirrhosis | 19 | 1.22(0.74-1.91) | 0 | 0(0-3.16) | 5 | 1.02(0.33-2.38) | 7 | 1.31(0.53-2.7) | 7 | 1.7(0.68-3.49) | |
| Nephritis, Nephrotic Syndrome and Nephrosis | 6 | 2.34(0.86-5.1) | 0 | 0(0-19.6) | 3 | 3.72(0.77-10.88) | 2 | 2.28(0.28-8.24) | 1 | 1.45(0.04-8.1) | |
| Symptoms, Signs and Ill-Defined Conditions | 9 | 1.88(0.86-3.56) | 2 | 4.68(0.57-16.92) | 4 | 2.38(0.65-6.08) | 2 | 1.26(0.15-4.57) | 1 | 0.91(0.02-5.06) | |
| Accidents and Adverse Effects | 64 | 1.05(0.81-1.34) | 11 | 2.52^#^(1.26-4.51) | 19 | 1.01(0.61-1.57) | 17 | 0.81(0.47-1.3) | 17 | 1.01(0.59-1.61) | |
| Suicide and Self-Inflicted Injury | 22 | 0.79(0.49-1.19) | 0 | 0(0-1.83) | 8 | 0.92(0.4-1.81) | 7 | 0.72(0.29-1.48) | 7 | 0.93(0.37-1.91) | |
| Homicide and Legal Intervention | 6 | 0.91(0.33-1.99) | 0 | 0(0-7.22) | 1 | 0.47(0.01-2.61) | 3 | 1.35(0.28-3.94) | 2 | 1.17(0.14-4.23) | |
| Other Cause of Death | 46 | 1.28(0.94-1.71) | 9 | 3.34^#^(1.53-6.34) | 17 | 1.49(0.87-2.39) | 11 | 0.9(0.45-1.6) | 9 | 0.95(0.44-1.81) | |

**1** number of cancer patients who died due to each cause of death

**2** standardized mortality rate

**3** 95% Confidence interval

**^#^** P value less than .05

| Supporting Table 5. Cause of death for testicular cancer patients 50+ years. | | | | | | | | | | |  |
| --- | --- | --- | --- | --- | --- | --- | --- | --- | --- | --- | --- |
|  | Total | | <1 years | | 1-5 years | | 5-10 years | | >10 years | | |
| Selected Events | Observed^1^ | SMR^2^  (95% CI^3^) | Observed | SMR | Observed | SMR | Observed | SMR | Observed | SMR | |
| All Causes of Death | 1,193 | 1.36^#^(1.29-1.44) | 266 | 5.14^#^(4.54-5.79) | 309 | 1.37^#^(1.22-1.53) | 277 | 0.98(0.87-1.1) | 341 | 1.08(0.97-1.2) | |
| All Malignant Cancers | 594 | 2.49^#^(2.29-2.7) | 199 | 14.00^#^(12.12-16.09) | 188 | 3.04^#^(2.62-3.51) | 93 | 1.2(0.97-1.47) | 114 | 1.34^#^(1.1-1.6) | |
| In situ, benign or unknown behavior neoplasm | 11 | 2.44^#^(1.22-4.36) | 3 | 10.81^#^(2.23-31.6) | 3 | 2.5(0.52-7.3) | 3 | 2.05(0.42-6) | 2 | 1.27(0.15-4.58) | |
| Septicemia | 15 | 1.3(0.73-2.15) | 5 | 7.53^#^(2.45-17.58) | 2 | 0.69(0.08-2.49) | 5 | 1.35(0.44-3.15) | 3 | 0.7(0.15-2.06) | |
| Other Infectious and Parasitic Diseases including HIV | 15 | 1.01(0.57-1.67) | 0 | 0(0-4.26) | 3 | 0.77(0.16-2.24) | 7 | 1.41(0.57-2.9) | 5 | 0.99(0.32-2.31) | |
| Diabetes Mellitus | 26 | 0.85(0.56-1.25) | 3 | 1.73(0.36-5.05) | 2 | 0.26^#^(0.03-0.95) | 6 | 0.61(0.23-1.34) | 15 | 1.31(0.74-2.17) | |
| Alzheimers | 8 | 0.88(0.38-1.74) | 0 | 0(0-6.16) | 4 | 1.55(0.42-3.98) | 1 | 0.34(0.01-1.91) | 3 | 1.01(0.21-2.96) | |
| Diseases of Heart | 192 | 0.88(0.76-1.01) | 18 | 1.32(0.79-2.09) | 40 | 0.69^#^(0.49-0.94) | 69 | 0.98(0.77-1.24) | 65 | 0.85(0.65-1.08) | |
| Hypertension without Heart Disease | 11 | 1.43(0.71-2.55) | 0 | 0(0-8.76) | 1 | 0.53(0.01-2.97) | 5 | 2.04(0.66-4.75) | 5 | 1.69(0.55-3.95) | |
| Cerebrovascular Diseases | 18 | 0.58^#^(0.34-0.92) | 3 | 1.48(0.31-4.34) | 4 | 0.48(0.13-1.22) | 4 | 0.41(0.11-1.04) | 7 | 0.66(0.26-1.35) | |
| Atherosclerosis | 2 | 1.44(0.17-5.2) | 1 | 9.01(0.23-50.19) | 1 | 2.35(0.06-13.08) | 0 | 0(0-8.49) | 0 | 0(0-8.83) | |
| Aortic Aneurysm and Dissection | 1 | 0.24(0.01-1.32) | 1 | 3.49(0.09-19.44) | 0 | 0(0-3.14) | 0 | 0(0-2.75) | 0 | 0(0-2.6) | |
| Other Diseases of Arteries, Arterioles, Capillaries | 3 | 1.17(0.24-3.42) | 0 | 0(0-23.3) | 1 | 1.49(0.04-8.3) | 1 | 1.23(0.03-6.84) | 1 | 1.09(0.03-6.05) | |
| Pneumonia and Influenza | 14 | 1.03(0.56-1.73) | 3 | 3.37(0.69-9.84) | 2 | 0.54(0.07-1.95) | 5 | 1.15(0.37-2.7) | 4 | 0.86(0.23-2.2) | |
| Chronic Obstructive Pulmonary Disease and Allied Cond | 39 | 0.93(0.66-1.28) | 3 | 1.2(0.25-3.5) | 2 | 0.19^#^(0.02-0.67) | 15 | 1.13(0.63-1.86) | 19 | 1.26(0.76-1.97) | |
| Stomach and Duodenal Ulcers | 0 | 0(0-3.06) | 0 | 0(0-49.22) | 0 | 0(0-11.67) | 0 | 0(0-9.59) | 0 | 0(0-8.55) | |
| Chronic Liver Disease and Cirrhosis | 29 | 0.97(0.65-1.39) | 0 | 0(0-2.38) | 7 | 0.98(0.39-2.02) | 5 | 0.51(0.17-1.19) | 17 | 1.47(0.86-2.36) | |
| Nephritis, Nephrotic Syndrome and Nephrosis | 12 | 0.93(0.48-1.63) | 2 | 2.53(0.31-9.13) | 4 | 1.17(0.32-3) | 2 | 0.48(0.06-1.75) | 4 | 0.88(0.24-2.26) | |
| Symptoms, Signs and Ill-Defined Conditions | 12 | 1.45(0.75-2.54) | 2 | 4(0.48-14.47) | 4 | 1.82(0.5-4.66) | 5 | 1.86(0.6-4.34) | 1 | 0.35(0.01-1.94) | |
| Accidents and Adverse Effects | 38 | 0.70^#^(0.5-0.96) | 3 | 1.07(0.22-3.13) | 11 | 0.85(0.42-1.52) | 6 | 0.34^#^(0.13-0.75) | 18 | 0.86(0.51-1.36) | |
| Suicide and Self-Inflicted Injury | 25 | 1.03(0.67-1.52) | 2 | 1.6(0.19-5.79) | 4 | 0.69(0.19-1.76) | 7 | 0.88(0.35-1.81) | 12 | 1.3(0.67-2.28) | |
| Homicide and Legal Intervention | 4 | 1.14(0.31-2.92) | 0 | 0(0-19.11) | 1 | 1.15(0.03-6.38) | 2 | 1.75(0.21-6.33) | 1 | 0.77(0.02-4.27) | |
| Other Cause of Death | 121 | 1.11(0.92-1.33) | 17 | 2.76^#^(1.61-4.42) | 25 | 0.91(0.59-1.34) | 34 | 0.97(0.67-1.35) | 45 | 1.12(0.82-1.5) | |

**1** number of cancer patients who died due to each cause of death

**2** standardized mortality rate

**3** 95% Confidence interval

**^#^** P value less than .05

| Supporting Table 6. Cause of death for testicular cancer in white. | | | | | | | | | | |  |
| --- | --- | --- | --- | --- | --- | --- | --- | --- | --- | --- | --- |
|  | Total | | <1 years | | 1-5 years | | 5-10 years | | >10 years | | |
| Selected Events | Observed^1^ | SMR^2^  (95% CI^3^) | Observed | SMR | Observed | SMR | Observed | SMR | Observed | SMR | |
| All Causes of Death | 2,861 | 2.12^#^(2.04-2.2) | 801 | 7.83^#^(7.3-8.39) | 1,124 | 2.74^#^(2.58-2.9) | 489 | 1.12^#^(1.02-1.22) | 447 | 1.12^#^(1.02-1.23) | |
| All Malignant Cancers | 1,772 | 6.23^#^(5.94-6.53) | 628 | 33.53^#^(30.96-36.26) | 806 | 10.17^#^(9.48-10.9) | 182 | 1.96^#^(1.68-2.26) | 156 | 1.67^#^(1.42-1.95) | |
| In situ, benign or unknown behavior neoplasm | 27 | 4.65^#^(3.06-6.77) | 8 | 19.06^#^(8.23-37.55) | 10 | 5.81^#^(2.79-10.69) | 6 | 3.18^#^(1.17-6.93) | 3 | 1.68(0.35-4.92) | |
| Septicemia | 26 | 1.77^#^(1.16-2.59) | 11 | 11.48^#^(5.73-20.55) | 5 | 1.23(0.4-2.87) | 5 | 1.05(0.34-2.44) | 5 | 1.02(0.33-2.39) | |
| Other Infectious and Parasitic Diseases including HIV | 33 | 1.29(0.89-1.82) | 5 | 2.29(0.74-5.35) | 10 | 1.18(0.56-2.16) | 11 | 1.32(0.66-2.37) | 7 | 1.08(0.43-2.22) | |
| Diabetes Mellitus | 36 | 0.92(0.64-1.27) | 4 | 1.59(0.43-4.07) | 8 | 0.74(0.32-1.46) | 8 | 0.62(0.27-1.23) | 16 | 1.21(0.69-1.97) | |
| Alzheimers | 7 | 0.8(0.32-1.66) | 0 | 0(0-6.51) | 4 | 1.61(0.44-4.13) | 1 | 0.35(0.01-1.97) | 2 | 0.71(0.09-2.55) | |
| Diseases of Heart | 246 | 0.88(0.78-1) | 31 | 1.63^#^(1.1-2.31) | 61 | 0.76^#^(0.58-0.98) | 82 | 0.9(0.72-1.12) | 72 | 0.81(0.64-1.03) | |
| Hypertension without Heart Disease | 10 | 1.13(0.54-2.09) | 0 | 0(0-7.25) | 1 | 0.44(0.01-2.46) | 5 | 1.74(0.57-4.07) | 4 | 1.26(0.34-3.23) | |
| Cerebrovascular Diseases | 25 | 0.68^#^(0.44-1) | 7 | 2.71^#^(1.09-5.58) | 7 | 0.66(0.26-1.35) | 4 | 0.34^#^(0.09-0.86) | 7 | 0.6(0.24-1.23) | |
| Atherosclerosis | 1 | 0.67(0.02-3.73) | 0 | 0(0-30.39) | 1 | 2.14(0.05-11.9) | 0 | 0(0-7.84) | 0 | 0(0-8.54) | |
| Aortic Aneurysm and Dissection | 2 | 0.33(0.04-1.2) | 2 | 4.42(0.54-15.97) | 0 | 0(0-2.02) | 0 | 0(0-1.89) | 0 | 0(0-2.07) | |
| Other Diseases of Arteries, Arterioles, Capillaries | 5 | 1.67(0.54-3.9) | 0 | 0(0-18.59) | 1 | 1.21(0.03-6.72) | 1 | 1.04(0.03-5.81) | 3 | 2.99(0.62-8.73) | |
| Pneumonia and Influenza | 21 | 1.21(0.75-1.85) | 4 | 3.15(0.86-8.06) | 7 | 1.35(0.54-2.78) | 5 | 0.89(0.29-2.08) | 5 | 0.94(0.31-2.2) | |
| Chronic Obstructive Pulmonary Disease and Allied Cond | 37 | 0.84(0.59-1.15) | 3 | 1.09(0.23-3.2) | 2 | 0.17^#^(0.02-0.61) | 16 | 1.13(0.64-1.83) | 16 | 1.03(0.59-1.68) | |
| Stomach and Duodenal Ulcers | 1 | 0.63(0.02-3.49) | 0 | 0(0-33.2) | 1 | 2.18(0.06-12.13) | 0 | 0(0-7.14) | 0 | 0(0-7.25) | |
| Chronic Liver Disease and Cirrhosis | 57 | 1.17(0.89-1.52) | 2 | 0.65(0.08-2.34) | 16 | 1.19(0.68-1.94) | 16 | 0.99(0.57-1.61) | 23 | 1.45(0.92-2.17) | |
| Nephritis, Nephrotic Syndrome and Nephrosis | 17 | 1.15(0.67-1.84) | 2 | 2.05(0.25-7.42) | 6 | 1.44(0.53-3.14) | 4 | 0.83(0.23-2.13) | 5 | 1.03(0.33-2.41) | |
| Symptoms, Signs and Ill-Defined Conditions | 40 | 2.26^#^(1.62-3.08) | 8 | 4.79^#^(2.07-9.44) | 19 | 3.08^#^(1.86-4.82) | 11 | 1.98(0.99-3.55) | 2 | 0.47(0.06-1.68) | |
| Accidents and Adverse Effects | 175 | 0.83^#^(0.71-0.96) | 38 | 1.84^#^(1.3-2.53) | 59 | 0.78(0.59-1) | 39 | 0.58^#^(0.41-0.79) | 39 | 0.81(0.57-1.11) | |
| Suicide and Self-Inflicted Injury | 83 | 0.91(0.72-1.12) | 4 | 0.46(0.13-1.18) | 32 | 0.99(0.68-1.4) | 24 | 0.81(0.52-1.21) | 23 | 1.09(0.69-1.64) | |
| Homicide and Legal Intervention | 18 | 0.81(0.48-1.28) | 1 | 0.37(0.01-2.08) | 6 | 0.67(0.24-1.45) | 7 | 1.05(0.42-2.16) | 4 | 1.03(0.28-2.64) | |
| Other Cause of Death | 216 | 1.34^#^(1.17-1.53) | 42 | 3.67^#^(2.65-4.96) | 60 | 1.27(0.97-1.63) | 59 | 1.13(0.86-1.45) | 55 | 1.1(0.83-1.43) | |

**1** number of cancer patients who died due to each cause of death

**2** standardized mortality rate

**3** 95% Confidence interval

**^#^** P value less than .05

| Supporting Table 7. Cause of death for testicular cancer in black. | | | | | | | | | | |  |
| --- | --- | --- | --- | --- | --- | --- | --- | --- | --- | --- | --- |
|  | Total | | <1 years | | 1-5 years | | 5-10 years | | >10 years | | |
| Selected Events | Observed^1^ | SMR^2^  (95% CI^3^) | Observed | SMR | Observed | SMR | Observed | SMR | Observed | SMR | |
| All Causes of Death | 185 | 3.04^#^(2.62-3.51) | 75 | 13.33^#^(10.49-16.71) | 58 | 2.88^#^(2.18-3.72) | 26 | 1.36(0.89-1.99) | 26 | 1.62^#^(1.06-2.38) | |
| All Malignant Cancers | 111 | 9.83^#^(8.09-11.84) | 54 | 58.71^#^(44.11-76.6) | 42 | 12.21^#^(8.8-16.5) | 7 | 1.92(0.77-3.95) | 8 | 2.44^#^(1.05-4.8) | |
| In situ, benign or unknown behavior neoplasm | 2 | 10.43^#^(1.26-37.67) | 0 | 0(0-216.77) | 2 | 32.63^#^(3.95-117.86) | 0 | 0(0-61.12) | 0 | 0(0-69.45) | |
| Septicemia | 1 | 1.03(0.03-5.75) | 0 | 0(0-44.88) | 0 | 0(0-12.21) | 1 | 3.23(0.08-17.99) | 0 | 0(0-13.38) | |
| Other Infectious and Parasitic Diseases including HIV | 8 | 2.77^#^(1.19-5.45) | 1 | 3.1(0.08-17.3) | 3 | 2.66(0.55-7.77) | 3 | 3.36(0.69-9.81) | 1 | 1.82(0.05-10.15) | |
| Diabetes Mellitus | 5 | 2.02(0.66-4.72) | 0 | 0(0-18.72) | 0 | 0(0-4.92) | 3 | 3.78(0.78-11.05) | 2 | 2.74(0.33-9.89) | |
| Alzheimers | 1 | 5.35(0.14-29.8) | 0 | 0(0-181.41) | 1 | 16.7(0.42-93.06) | 0 | 0(0-80.9) | 0 | 0(0-60.31) | |
| Diseases of Heart | 17 | 1.2(0.7-1.93) | 3 | 2.55(0.53-7.47) | 3 | 0.68(0.14-2) | 6 | 1.33(0.49-2.89) | 5 | 1.23(0.4-2.88) | |
| Hypertension without Heart Disease | 1 | 1.12(0.03-6.26) | 0 | 0(0-52.64) | 0 | 0(0-13.69) | 0 | 0(0-12.96) | 1 | 3.77(0.1-21) | |
| Cerebrovascular Diseases | 2 | 0.78(0.1-2.83) | 1 | 4.62(0.12-25.76) | 0 | 0(0-4.66) | 0 | 0(0-4.54) | 1 | 1.37(0.03-7.65) | |
| Atherosclerosis | 1 | 18.92(0.48-105.42) | 1 | 187.85^#^(4.76-1046.61) | 0 | 0(0-220.3) | 0 | 0(0-223.26) | 0 | 0(0-258.71) | |
| Aortic Aneurysm and Dissection | 0 | 0(0-11.8) | 0 | 0(0-134.85) | 0 | 0(0-36) | 0 | 0(0-36.5) | 0 | 0(0-45.17) | |
| Other Diseases of Arteries, Arterioles, Capillaries | 0 | 0(0-22.61) | 0 | 0(0-272.62) | 0 | 0(0-75.13) | 0 | 0(0-71.62) | 0 | 0(0-75.26) | |
| Pneumonia and Influenza | 2 | 2.51(0.3-9.07) | 2 | 27.78^#^(3.36-100.36) | 0 | 0(0-14.55) | 0 | 0(0-14.86) | 0 | 0(0-16.55) | |
| Chronic Obstructive Pulmonary Disease and Allied Cond | 2 | 1.54(0.19-5.57) | 0 | 0(0-33.25) | 0 | 0(0-9.32) | 1 | 2.45(0.06-13.65) | 1 | 2.62(0.07-14.58) | |
| Stomach and Duodenal Ulcers | 0 | 0(0-47.57) | 0 | 0(0-531.26) | 0 | 0(0-146.28) | 0 | 0(0-149.53) | 0 | 0(0-178.05) | |
| Chronic Liver Disease and Cirrhosis | 1 | 0.97(0.02-5.4) | 0 | 0(0-45.7) | 0 | 0(0-11.57) | 0 | 0(0-11.07) | 1 | 3.34(0.08-18.58) | |
| Nephritis, Nephrotic Syndrome and Nephrosis | 0 | 0(0-2.78) | 0 | 0(0-33.64) | 0 | 0(0-9.01) | 0 | 0(0-8.73) | 0 | 0(0-9.57) | |
| Symptoms, Signs and Ill-Defined Conditions | 2 | 2.4(0.29-8.65) | 2 | 21.90^#^(2.65-79.1) | 0 | 0(0-11.96) | 0 | 0(0-14.76) | 0 | 0(0-19.9) | |
| Accidents and Adverse Effects | 12 | 1.89(0.98-3.31) | 8 | 12.53^#^(5.41-24.69) | 1 | 0.44(0.01-2.47) | 1 | 0.51(0.01-2.86) | 2 | 1.33(0.16-4.82) | |
| Suicide and Self-Inflicted Injury | 3 | 2.33(0.48-6.8) | 0 | 0(0-24.26) | 2 | 3.94(0.48-14.25) | 0 | 0(0-9.42) | 1 | 4.18(0.11-23.28) | |
| Homicide and Legal Intervention | 3 | 0.65(0.13-1.9) | 0 | 0(0-5.86) | 1 | 0.51(0.01-2.83) | 2 | 1.51(0.18-5.44) | 0 | 0(0-5.33) | |
| Other Cause of Death | 11 | 1.6(0.8-2.86) | 3 | 4.92^#^(1.01-14.37) | 3 | 1.35(0.28-3.94) | 2 | 0.93(0.11-3.36) | 3 | 1.58(0.33-4.63) | |

**1** number of cancer patients who died due to each cause of death

**2** standardized mortality rate

**3** 95% Confidence interval

**^#^** P value less than .05

| Supporting Table 8. Cause of death for testicular cancer in other. | | | | | | | | | | |  |
| --- | --- | --- | --- | --- | --- | --- | --- | --- | --- | --- | --- |
|  | Total | | <1 years | | 1-5 years | | 5-10 years | | >10 years | | |
| Selected Events | Observed^1^ | SMR^2^  (95% CI^3^) | Observed | SMR | Observed | SMR | Observed | SMR | Observed | SMR | |
| All Causes of Death | 174 | 5.08^#^(4.36-5.9) | 56 | 18.48^#^(13.96-23.99) | 83 | 7.37^#^(5.87-9.14) | 16 | 1.51(0.86-2.45) | 19 | 2.04^#^(1.23-3.18) | |
| All Malignant Cancers | 122 | 16.38^#^(13.6-19.56) | 45 | 79.17^#^(57.75-105.94) | 64 | 28.36^#^(21.84-36.21) | 6 | 2.55(0.93-5.54) | 7 | 3.09^#^(1.24-6.36) | |
| In situ, benign or unknown behavior neoplasm | 0 | 0(0-24.26) | 0 | 0(0-287.33) | 0 | 0(0-75.57) | 0 | 0(0-77.33) | 0 | 0(0-86.41) | |
| Septicemia | 2 | 5.48(0.66-19.81) | 2 | 68.19^#^(8.26-246.33) | 0 | 0(0-32.46) | 0 | 0(0-32.22) | 0 | 0(0-34.38) | |
| Other Infectious and Parasitic Diseases including HIV | 1 | 1.55(0.04-8.65) | 0 | 0(0-63.7) | 0 | 0(0-16.69) | 1 | 4.9(0.12-27.32) | 0 | 0(0-22.88) | |
| Diabetes Mellitus | 2 | 1.49(0.18-5.38) | 0 | 0(0-38.34) | 0 | 0(0-9.52) | 0 | 0(0-8.65) | 2 | 4.62(0.56-16.69) | |
| Alzheimers | 1 | 4.18(0.11-23.3) | 0 | 0(0-207.66) | 0 | 0(0-60.14) | 0 | 0(0-50.04) | 1 | 11.59(0.29-64.56) | |
| Diseases of Heart | 5 | 0.72(0.23-1.68) | 2 | 3.61(0.44-13.02) | 2 | 0.94(0.11-3.38) | 1 | 0.46(0.01-2.55) | 0 | 0(0-1.79) | |
| Hypertension without Heart Disease | 0 | 0(0-10.73) | 0 | 0(0-154.88) | 0 | 0(0-39.16) | 0 | 0(0-34.3) | 0 | 0(0-31.24) | |
| Cerebrovascular Diseases | 1 | 0.62(0.02-3.46) | 0 | 0(0-28.16) | 1 | 2(0.05-11.14) | 0 | 0(0-7.32) | 0 | 0(0-7.74) | |
| Atherosclerosis | 0 | 0(0-100.03) | 0 | 0(0-1140.08) | 0 | 0(0-304.14) | 0 | 0(0-328.26) | 0 | 0(0-358.94) | |
| Aortic Aneurysm and Dissection | 1 | 5.42(0.14-30.22) | 0 | 0(0-229.45) | 1 | 16.08(0.41-89.59) | 0 | 0(0-64.18) | 0 | 0(0-75.86) | |
| Other Diseases of Arteries, Arterioles, Capillaries | 0 | 0(0-62.1) | 0 | 0(0-771.98) | 0 | 0(0-200.65) | 0 | 0(0-200.5) | 0 | 0(0-206.73) | |
| Pneumonia and Influenza | 1 | 1.43(0.04-7.97) | 0 | 0(0-63.67) | 1 | 4.65(0.12-25.91) | 0 | 0(0-16.87) | 0 | 0(0-17.77) | |
| Chronic Obstructive Pulmonary Disease and Allied Cond | 2 | 2.63(0.32-9.49) | 0 | 0(0-59.4) | 0 | 0(0-15.73) | 0 | 0(0-15.8) | 2 | 8.64^#^(1.05-31.19) | |
| Stomach and Duodenal Ulcers | 0 | 0(0-62.72) | 0 | 0(0-772.02) | 0 | 0(0-203.32) | 0 | 0(0-198.78) | 0 | 0(0-212.8) | |
| Chronic Liver Disease and Cirrhosis | 1 | 0.77(0.02-4.3) | 0 | 0(0-35.88) | 0 | 0(0-8.92) | 0 | 0(0-8.76) | 1 | 2.8(0.07-15.61) | |
| Nephritis, Nephrotic Syndrome and Nephrosis | 1 | 2.02(0.05-11.28) | 0 | 0(0-98.93) | 1 | 6.78(0.17-37.78) | 0 | 0(0-23.91) | 0 | 0(0-23.8) | |
| Symptoms, Signs and Ill-Defined Conditions | 2 | 5.01(0.61-18.1) | 0 | 0(0-84.59) | 2 | 13.44^#^(1.63-48.55) | 0 | 0(0-31.13) | 0 | 0(0-41.77) | |
| Accidents and Adverse Effects | 17 | 3.94^#^(2.3-6.31) | 3 | 6.06^#^(1.25-17.7) | 6 | 3.55^#^(1.3-7.73) | 4 | 3.12(0.85-7.99) | 4 | 4.73^#^(1.29-12.12) | |
| Suicide and Self-Inflicted Injury | 5 | 2.52(0.82-5.89) | 0 | 0(0-15.21) | 3 | 3.7(0.76-10.81) | 2 | 3.43(0.41-12.37) | 0 | 0(0-10.75) | |
| Homicide and Legal Intervention | 1 | 1.19(0.03-6.64) | 0 | 0(0-32.63) | 1 | 2.76(0.07-15.36) | 0 | 0(0-15.54) | 0 | 0(0-29.18) | |
| Other Cause of Death | 8 | 2.1(0.91-4.14) | 3 | 9.39^#^(1.94-27.44) | 1 | 0.83(0.02-4.63) | 2 | 1.68(0.2-6.09) | 2 | 1.83(0.22-6.59) | |

**1** number of cancer patients who died due to each cause of death

**2** standardized mortality rate

**3** 95% Confidence interval

**^#^** P value less than .05

| Supporting Table 9. Definition of each cause of death and corresponding codes in the ICD-10 of Diseases and Related Health. | | |
| --- | --- | --- |
| Non-Cancer Causes of Death | ICD-10 corresponding codes | Cause of death definition |
| In situ, benign or unknown behavior neoplasms | D00-D09 | In situ neoplasms |
|  | D10-D36 | Benign neoplasms |
|  | D37-D48 | Neoplasms of uncertain or unknown behavior |
| Septicemia | A40-A41 | Sepsis |
| Other Infectious Diseases |  |  |
| Diabetes Mellitus | E10-E14 | Diabetes mellitus |
| Alzheimer’s | G30 | Alzheimer disease |
| Diseases of Heart | I00-I02 | Acute rheumatic fever |
|  | I05-I09 | Chronic rheumatic heart diseases |
|  | I11 | Hypertensive heart disease |
|  | I13 | Hypertensive heart and renal disease |
|  | I20-I25 | Ischemic heart diseases |
|  | I26-I28 | Pulmonary heart disease and diseases of pulmonary circulation |
|  | I30-I32 | Diseases of pericardium |
|  | I33 | Acute and subacute endocarditis |
|  | I34-I39 | Nonrheumatic valve disorders |
|  | I40-I41 | Myocarditis |
|  | I42-I43 | Cardiomyopathy |
|  | I44-I45 | Conduction disorders |
|  | I46 | Cardiac arrest |
|  | I47-I49 | Arrythmias |
|  | I50 | Heart failure |
|  | I51 | Complications and ill-defined descriptions of heart disease |
| Hypertension without Heart Disease | I10 | Essential (primary) hypertension |
|  | I12 | Hypertensive renal disease |
| Cerebrovascular Diseases | I60-I62 | Nontraumatic intracranial haemorrhage |
|  | I63 | Cerebral infarction |
|  | I64 | Stroke, not specified as haemorrhage or infarction |
|  | I65-I66 | Occlusion and stenosis of precerebral/cerebral arteries, not resulting in cerebral infarction |
|  | I67-I69 | Other cerebrovascular diseases or Sequelae of cerebrovascular disease |
| Atherosclerosis | I70 | Atherosclerosis |
| Aortic Aneurysm and Dissection | I71 | Aortic Aneurysm and Dissection |
| Other Diseases of Arteries, Arterioles, Capillaries | I72-I73 | Other aneurysm and dissection or other peripheral vascular diseases |
|  | I74 | Arterial embolism and thrombosis |
|  | I77 | Other disorders of arteries and arterioles |
|  | I78 | Diseases of capillaries |
| Pneumonia and Influenza | J09-J18 | Influenza and pneumonia |
| Chronic Obstructive Pulmonary Disease | J40-J42 | Bronchitis |
|  | J43 | Emphysema |
|  | J44 | Other chronic obstructive pulmonary disease |
|  | J45-J46 | Asthma or Status asthmaticus |
|  | J47 | Bronchiectasis |
| Chronic Liver Disease and Cirrhosis | K70 | Alcoholic liver disease |
|  | K73 | Chronic hepatitis |
|  | K74 | Fibrosis and cirrhosis of liver |
| Nephritis, Nephrotic Syndrome and Nephrosis | N00-N07 | Glomerular diseases |
|  | N17-N19 | Renal failure |
|  | N25 | Disorders resulting from impaired renal tubular function |
|  | N26 | Unspecified contracted kidney |
|  | N27 | Small kidney of unknown cause |
| Symptoms, Signs and Ill-Defined Conditions | R00-R99 | Symptoms, signs, abnormal results of clinical or other investigative procedures, and ill-defined conditions regarding which no diagnosis classifiable elsewhere is recorded. |
| Accidents and Adverse Effects | V01-V99 | Transport accidents |
|  | W00-X59 | Other external causes of accidental injury |
|  | Y85-Y86 | Sequelae of transport accidents or other accidents |
| Suicide and Self-Inflicted Injury | X60-X84 | Intentional self-harm |
|  | Y87 | Sequelae of intentional self-harm, assault and events of undetermined intent |
| Other Cause of Death |  |  |

Supporting Table 10. Impact of year of diagnosis on different categories of COD.

| COD | Levene's Test | P Value |
| --- | --- | --- |
| All Malignant Cancers | 0.116977 | 0.031019 |
| Testis | 0.334139 | 0.31409 |
| other cancer | 0.073347 | 0.01653 |
| non cancer | 0.012174 | 0.069296 |
| Other Cause of Death | 0.110664 | 0.639354 |

Levene's Test: F test

Supporting Table 11.
